# Supplementary material for: H3.3 contributes to chromatin accessibility and transcription factor binding at promoter-proximal regulatory elements in embryonic stem cells
Source: Genome Biol. 2023 Feb 13;24:25. doi: 10.1186/s13059-023-02867-3 (PMC9926682; doi:10.1186/s13059-023-02867-3)
Supplement: Supplementary file 1 — Additional file 1: Fig. S1. Loss of H3.3 deposition modestly reduces chromatin accessibility at enhancers. Fig. S2. HIRA facilitates H3.3 deposition at enhancers and promoters. Fig. S3. Loss of HIRA-mediated H3.3 deposition reduces chromatin accessibility at promoters. Fig. S4. HIRA-dependent loss of H3.3 deposition alters promoter architecture. Fig. S5. H3.3-dependent promoters display hallmarks of active chromatin. Fig. S6. ATRX/DAXX-dependent loss of H3.3 deposition increases accessibility at IAPEz. Fig. S7. Reduced ATAC-seq footprinting in the absence of H3.3. Fig. S8. Loss of HIRA phenocopies promoter dysregulation. Fig. S9. Altered H3.3 deposition influences TF binding at repeat elements. Fig. S10. Changes in promoter architecture are not correlated with transcriptional changes in associated proteins. Fig. S11. Chromatin Landsacpe Is Dysregulated with H3.3 Loss. Fig. S12. Loss of H3.3 alters RNAPII activty. Fig. S13. Transcriptional changes associated with loss of H3.3 in ESCs and during differentiation. Fig. S14. Uncropped blots from Fig. 6. Fig. S15. Exogenously expressed TFs bind at both open and closed chromatin in WT ESCs. [file 13059_2023_2867_MOESM1_ESM.pdf]

H3.3 contributes to chromatin accessibility and transcription factor binding at promoter-proximal regulatory elements in embryonic stem cells

Authors

Amanuel Tafessu[1], Ryan O'Hara[1], Sara Martire[1], Altair L. Dube, Purbita Saha, Vincent U. Gant, Laura A. Banaszynski[2]*

Affiliation

Cecil H. and Ida Green Center for Reproductive Biology Sciences, Department of Obstetrics and Gynecology, Children's Medical Center Research Institute, Harold C. Simmons Comprehensive Cancer Center, Hamon Center for Regenerative Science and Medicine, University of Texas Southwestern Medical Center, Dallas, Texas 75390, USA.

[1]These authors contributed equally.

[2]@BanaszynskiLab

*Correspondence: Laura.Banaszynski@UTSouthwestern.edu

Supplementary figures and figure legends (S1-S15).

Fig. S1. Loss of H3.3 deposition modestly reduces chromatin accessibility at enhancers

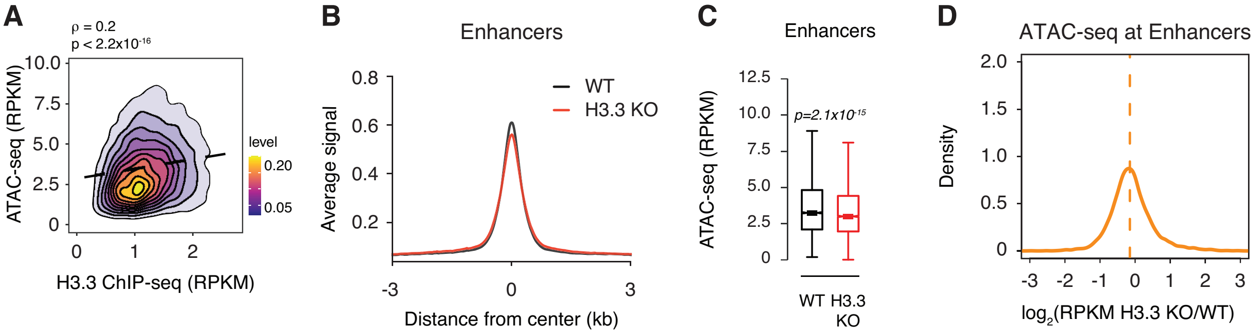

Fig. S1. Related to Figure 1. **Loss of H3.3 deposition modestly reduces chromatin accessibility at enhancers.**

A Correlation plot between ATAC-seq and H3.3 ChIP-seq at enhancers (n = 7,972) in ESCs.

B ATAC-seq average profiles at enhancers (n = 7,972) in WT and H3.3 KO ESCs.

C Boxplot showing ATAC-seq signal at enhancers (n = 7,972) in WT and H3.3 KO ESCs. The bottom and top of the boxes correspond to the 25th and 75th percentiles, and the internal band is the 50th percentile (median). The plot whiskers correspond to 1.5x interquartile range and outliers are excluded. P-values determined by Wilcoxon rank sum two-side test.

D Ratio (log2) of ATAC-seq signal at enhancers (n = 7,962) in WT and H3.3 KO ESCs. x axis values <0 indicate reduced accessibility in the absence of H3.3.

Fig. S2. HIRA facilitates H3.3 deposition at enhancers and promoters

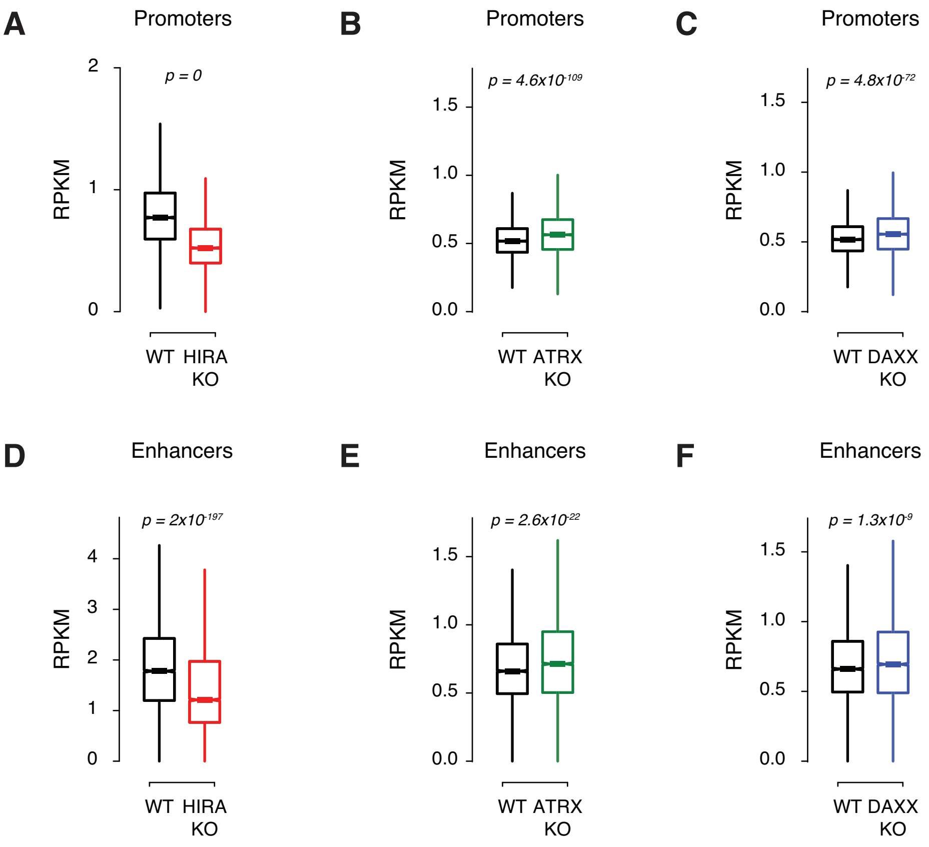

Fig. S2. Related to Figure 1. **HIRA facilitates H3.3 deposition at enhancers and promoters.**
A-C Box plot showing H3.3 ChIP-seq enrichment at active promoters (n =12,903) in WT and **(A)** HIRA KO, **(B)** ATRX KO, or **(C)** DAXX KO ESCs.
D-F Boxplot showing H3.3 ChIP-seq enrichment at enhancers (n = 7,972) in WT and **(D)** HIRA KO, **(E)** ATRX KO, or **(F)** DAXX KO ESCs.
The bottom and top of the boxes correspond to the 25th and 75th percentiles, and the internal band is the 50th percentile (median). The plot whiskers correspond to 1.5x interquartile range and outliers are excluded. P-values determined by Wilcoxon rank sum two-side test.

Fig. S3. Loss of HIRA-mediated H3.3 deposition reduces chromatin accessibility at promoters

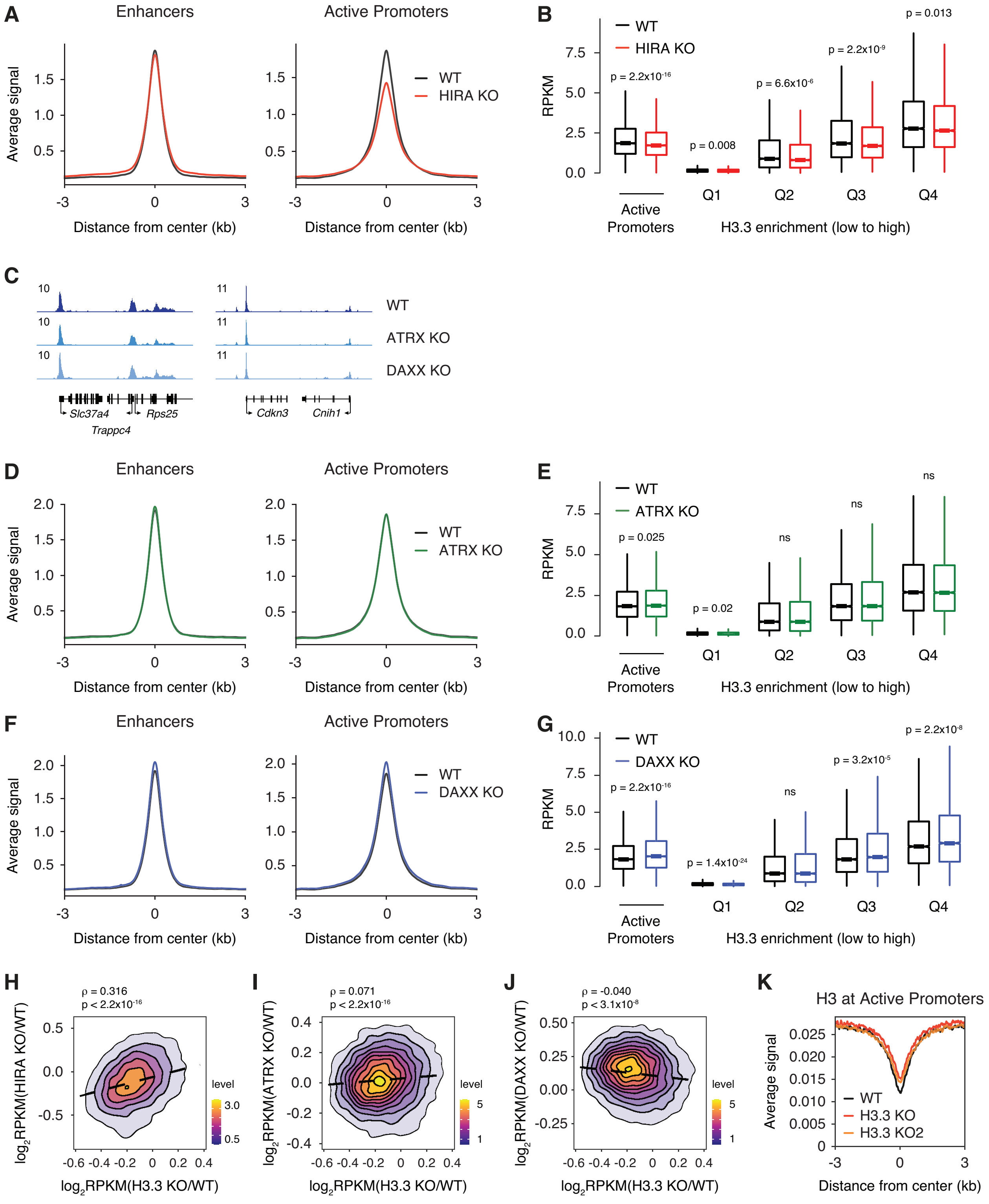

Fig. S3. Related to Figure 1. **Loss of HIRA-mediated H3.3 deposition reduces chromatin accessibility at promoters.**

A, D, F ATAC-seq average profiles at active promoters (n = 12,903, left) and enhancers (n = 7,972, right) in WT and **(A)** HIRA KO, **(D)** ATRX KO, or **(F)** DAXX KO ESCs.
B, E, G Boxplot showing ATAC-seq signal at active promoters (n = 12,903) and promoters binned by H3.3 enrichment in WT and **(B)** HIRA KO, **(E)** ATRX KO, or **(G)** DAXX KO ESCs. For all boxplots, the bottom and top of the boxes correspond to the 25th and 75th percentiles, and the internal band is the 50th percentile (median). The plot whiskers correspond to 1.5x interquartile range and outliers are excluded. P-values determined by Wilcoxon rank sum two-side test.
C Genome browser representations of ATAC-seq in WT, ATRX KO, and DAXX KO ESCs. The y-axis represents read density in reads per kilobase per million mapped reads (RPKM).
H-J Correlation plot between differential ATAC-seq signal in **(H)** HIRA KO, **(I)** ATRX KO, or **(J)** DAXX KO ESCs compared to WT ESCs (y-axis) and H3.3 KO compared to WT ESCs (x-axis) at active promoters (n = 12,903).
K Average profile of general H3 enrichment at active promoters (n = 12,903) in WT and two independent H3.3 KO ESC clones. 3 kb around the center of promoters are displayed.

Fig. S4. HIRA-dependent loss of H3.3 deposition alters promoter architecture

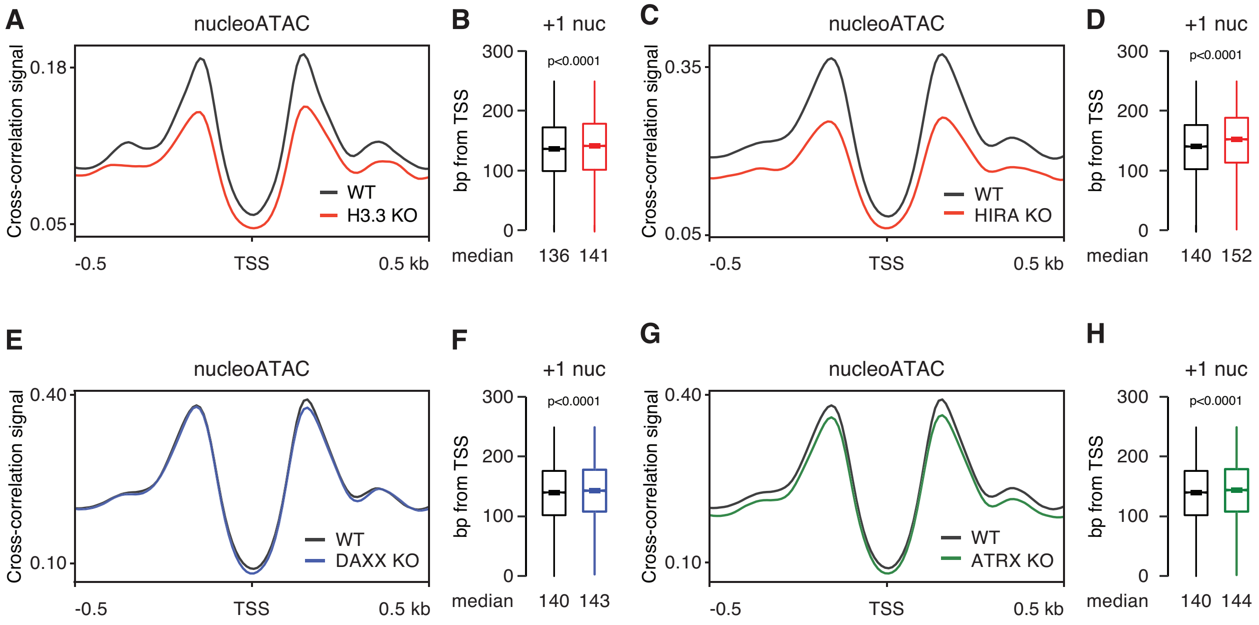

Fig. S4. Related to Figure 1. **HIRA-dependent loss of H3.3 deposition alters promoter architecture.**

A, C, E, G Positive NucleoATAC cross-correlation signal at the TSS of active genes (n = 12,903) in WT and **(A)** H3.3 KO, **(C)** HIRA KO, **(E)** DAXX KO, and **(G)** ATRX KO ESCs.

B, D, F, H Boxplot representing distribution of the +1 nucleosome from the TSS in WT and **(B)** H3.3 KO, **(D)** HIRA KO, **(F)** DAXX KO, and **(H)** ATRX KO ESCs. The bottom and top of the boxes correspond to the 25th and 75th percentiles, and the internal band is the 50th percentile (median). The plot whiskers correspond to 1.5x interquartile range and outliers are excluded. P-values determined by Wilcoxon rank sum two-side test.

Fig. S5. H3.3-dependent promoters display hallmarks of active chromatin

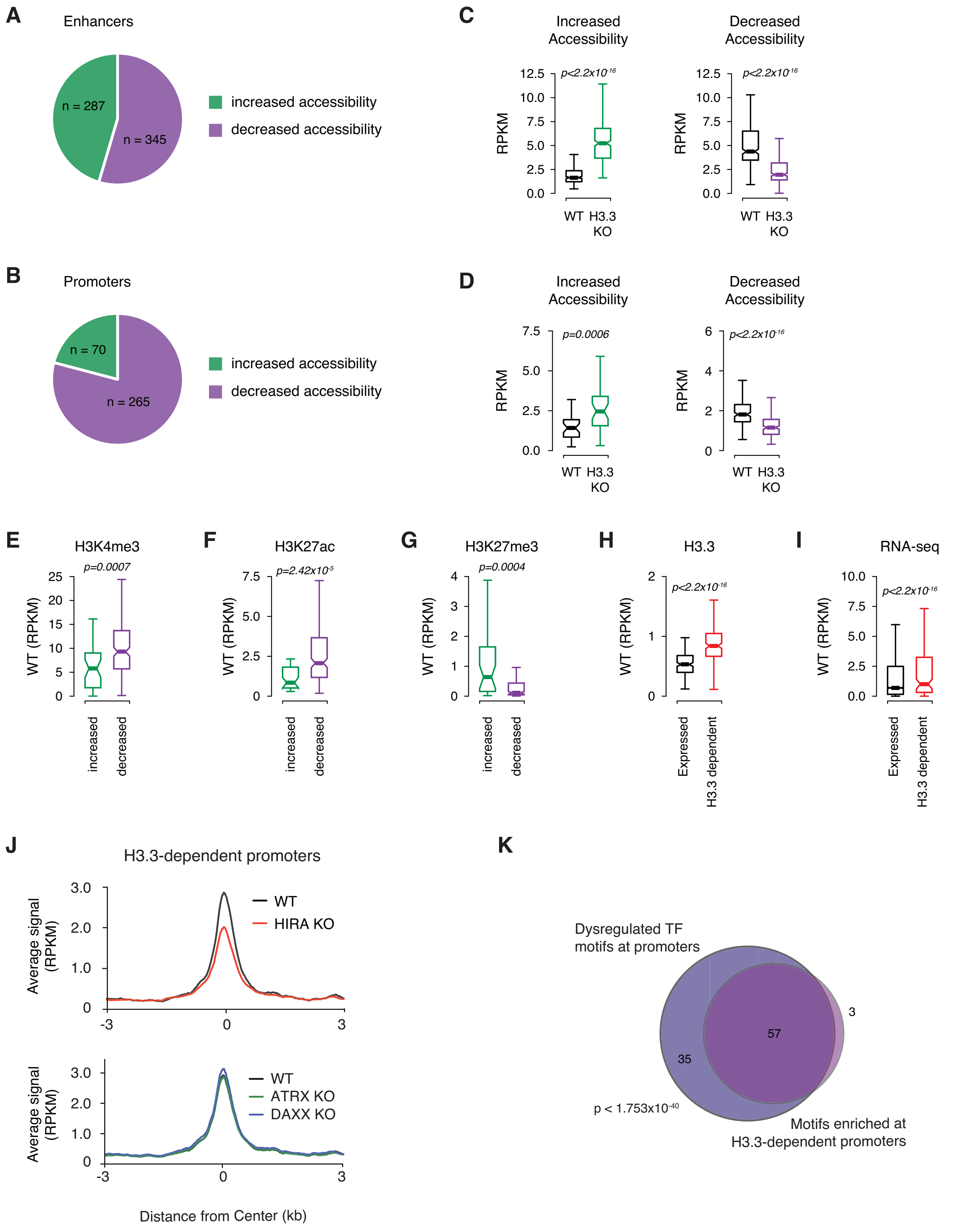

Fig. S5. Related to Figure 1. **H3.3-dependent promoters display hallmarks of active chromatin.**

A,B Representation of the number of differentially accessible **(A)** enhancers and **(B)** promoters in H3.3 KO compared to WT ESCs, determined using DiffBind.

C,D Boxplot showing ATAC-seq signal at differentially accessible **(C)** enhancers and **(D)** promoters in WT and H3.3 KO ESCs.

E-G Boxplot showing **(E)** H3K4me3, **(F)** H3K27ac, and **(G)** H3K27me3 ChIP-seq signal in WT ESCs at promoters with increased (n = 70) or decreased (n = 265) accessibility in H3.3 KO ESCs.

H, I Boxplot showing **(H)** H3.3 ChIP-seq signal and **(I)** RNA-seq expression at expressed genes (n = 12,903) and genes with H3.3-dependent promoters (n = 265) in WT ESCs.

For all box plots, the bottom and top of the boxes correspond to the 25th and 75th percentiles, and the internal band is the 50th percentile (median). The plot whiskers correspond to 1.5x interquartile range and outliers are excluded. P-values determined by Wilcoxon rank sum two-side test.

J ATAC-seq average profiles at H3.3-dependent promoters (n = 265) in WT and HIRA KO (top) and ATRX or DAXX KO (bottom) ESCs.

K Venn diagram representing overlap between the top dysregulated motifs at promoters in H3.3 KO ESCs classified based on Manhattan scores in the top 10% across all comparisons (i.e., WT vs H3.3 KO, HIRA KO, ATRX KO, or DAXX KO) and TF motifs enriched at H3.3-dependent promoters.

Fig. S6. ATRX/DAXX-dependent loss of H3.3 deposition increases accessibility at IAPEz

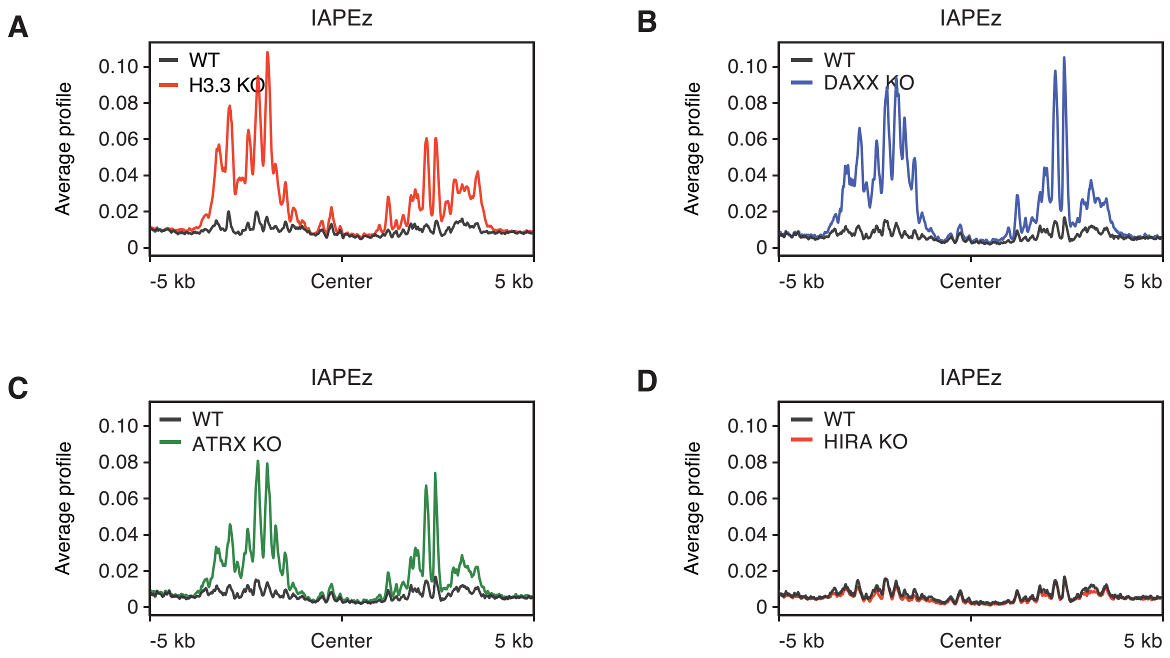

Fig. S6. Related to Figure 1. **ATRX/DAXX-dependent loss of H3.3 deposition increases accessibility at IAPEz.**
A-D ATAC-seq average profiles at IAPEz elements longer than 6 kb (n = 877) in WT and **(A)** H3.3 KO, **(B)** DAXX KO, **(C)** ATRX KO, or **(D)** HIRA KO ESCs.

Fig. S7. Reduced ATAC-seq footprinting in the absence of H3.3

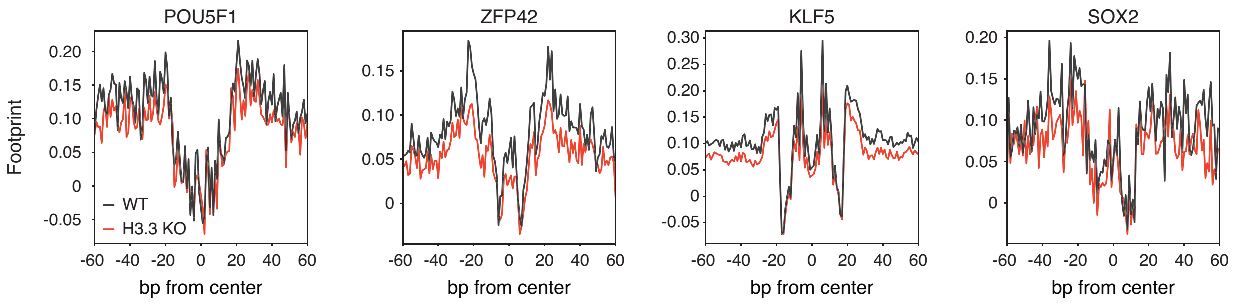

Fig. S7. Related to Figure 2. **Reduced ATAC-seq footprinting in the absence of H3.3.** Aggregated footprinting plots for representative promoter TF binding sites in WT and H3.3 KO ESCs.

Fig. S8. Loss of HIRA phenocopies promoter dysregulation

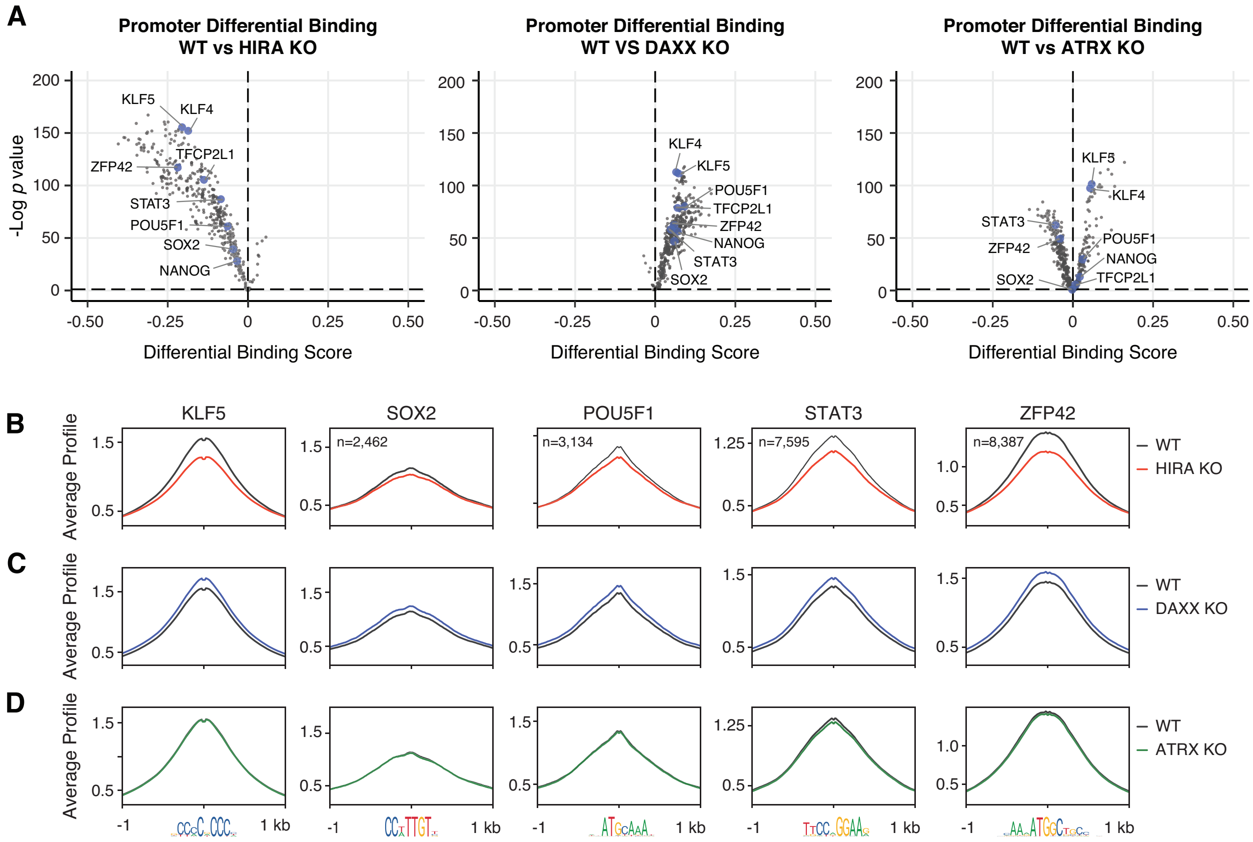

Fig. S8. Related to Figure 2. **Loss of HIRA phenocopies promoter dysregulation.**

A Pairwise comparison of TF activity at active promoters (n = 12,903) between WT and HIRA KO (left), DAXX KO (center), and ATRX KO (right) ESCs. Each TF is represented by a single circle (n = 395). TF motifs enriched in WT ESCs have negative differential binding scores and TF motifs enriched in chaperone KO ESCs have positive differential binding scores. Motifs for a subset of pluripotency-associated TFs are highlighted in blue.

B-D ATAC-seq average profiles at representative TF motifs at promoters in WT and **(B)** HIRA KO, **(C)** DAXX KO, and **(D)** ATRX KO ESCs. Data are centered on the motif and the number of motifs profiled are indicated.

Fig. S9. Altered H3.3 deposition influences TF binding at repeat elements

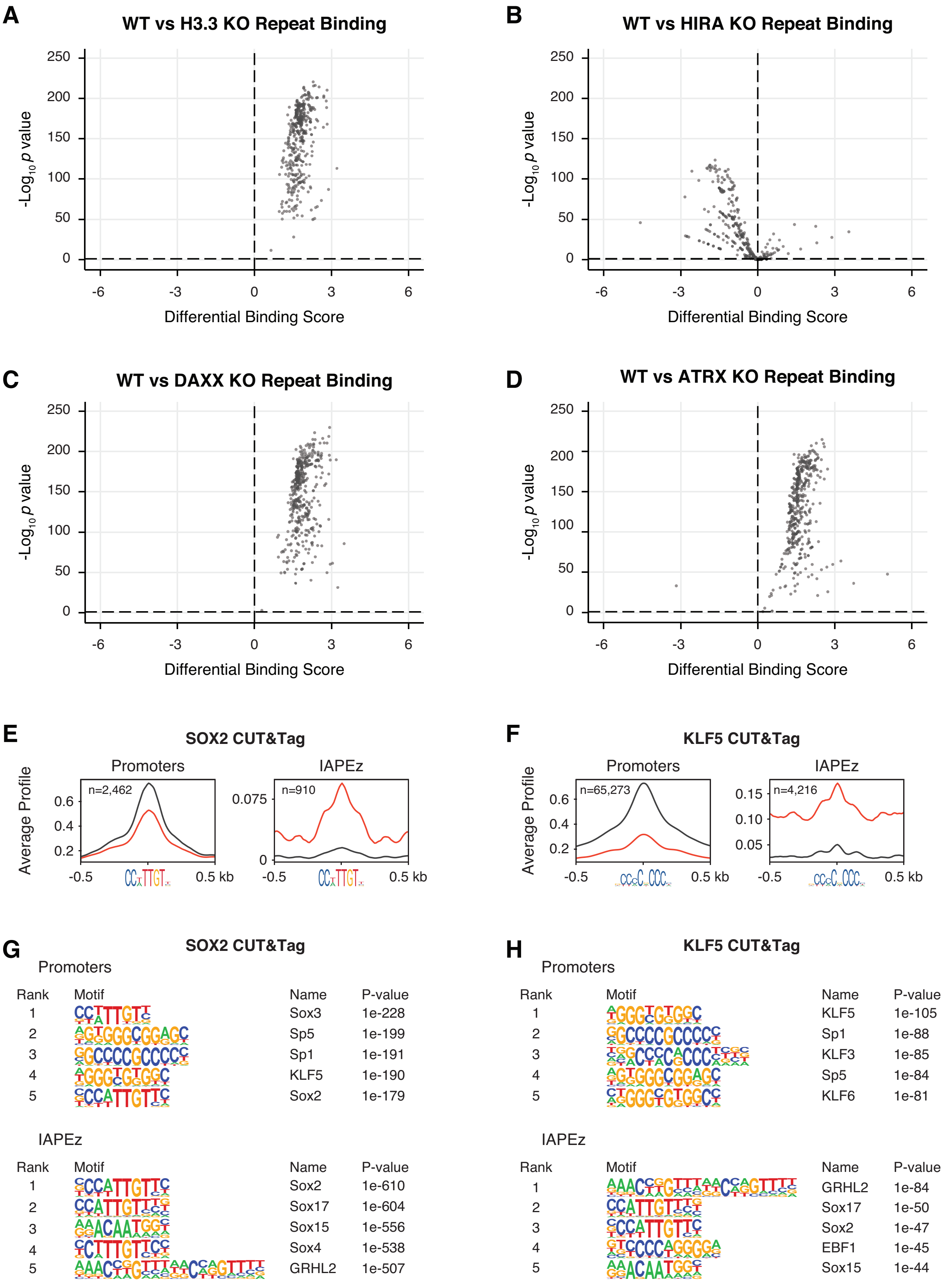

Fig. S9. Related to Figure 2. **Altered H3.3 deposition influences TF binding at repeat elements.**
A-D Pairwise comparison of TF activity at IAPEz repeat elements between WT and **(A)** H3.3 KO, **(B)** HIRA KO, **(C)** DAXX KO, and **(D)** ATRX KO ESCs. Each TF is represented by a single circle (n = 395). TF motifs enriched in WT ESCs have negative differential binding scores and TF motifs enriched in KO ESCs have positive differential binding scores.
E SOX2 CUT&Tag average profiles of SOX2 motifs at promoters (left) and IAPEz (right) in WT and H3.3 KO ESCs. Data are centered on the motif and the number of motifs profiled are indicated.
F KLF5 CUT&Tag average profiles of KLF5 motifs at promoters (left) and IAPEz (right) in WT and H3.3 KO ESCs. Data are centered on the motif and the number of motifs profiled are indicated.
G Motifs enriched at Promoters (top) and IAPEz (bottom) from SOX2 CUT&Tag data. Known motif analysis was performed using HOMER.
H Motifs enriched at Promoters (top) and IAPEz (bottom) from KLF5 CUT&Tag data. Known motif analysis was performed using HOMER.

Fig. S10. Changes in promoter architecture are not correlated with transcriptional changes in associated proteins

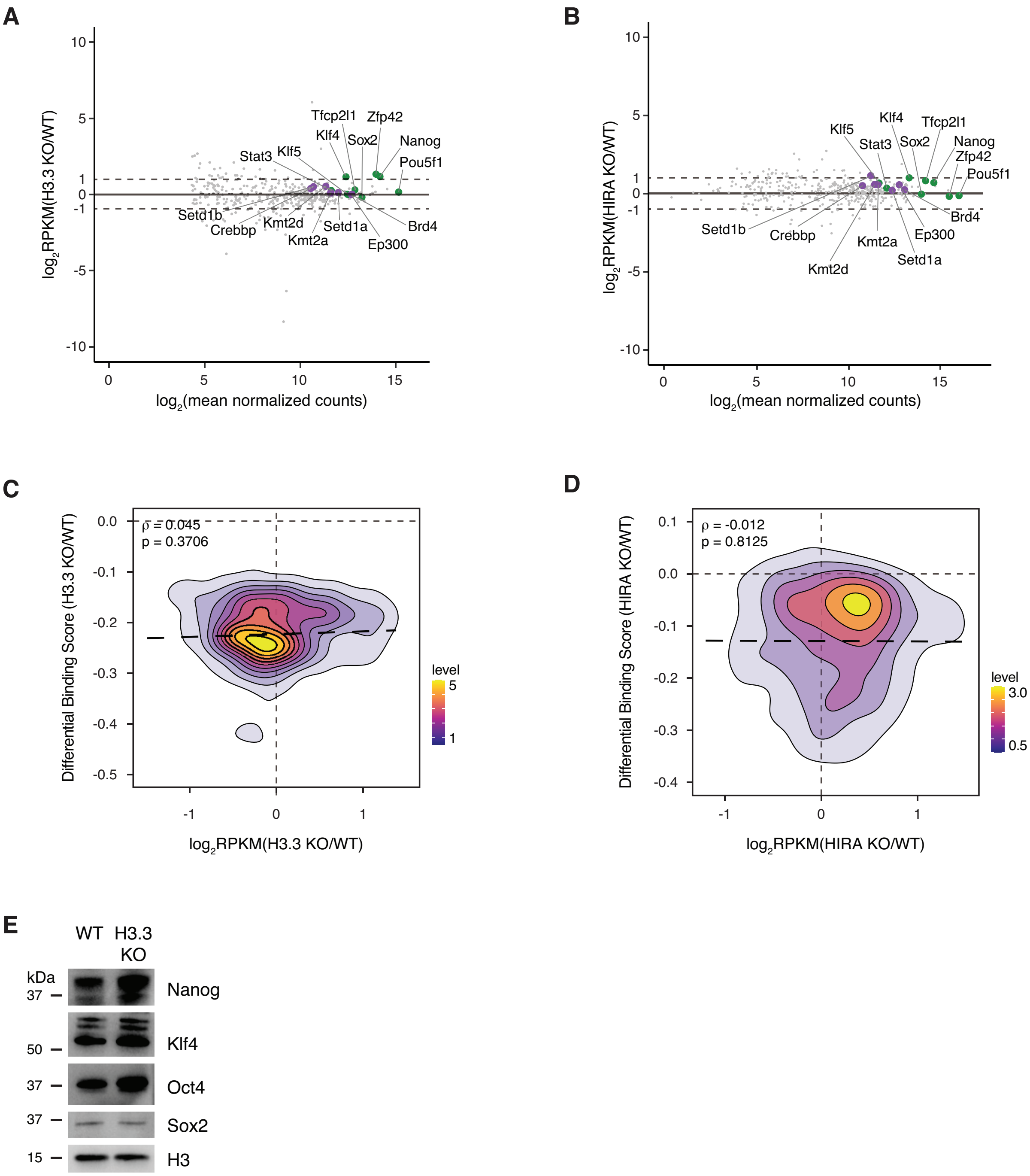

Fig. S10. Related to Figure 2. **Changes in promoter architecture are not correlated with transcriptional changes in associated proteins.**

A, B MA plot representing all TFs represented in motif analysis and select chromatin-associated proteins. Mean expression across compared samples is represented on the x-axis and differential expression between WT and **(A)** H3.3 KO or **(B)** HIRA KO ESCs is represented on

the y-axis. Highlighted TFs are labeled green and all chromatin-associated proteins are labeled purple.
C, D Correlation plot between differential expression in WT and **(C)** H3.3 KO or **(D)** HIRA KO ESCs and differential TF binding score in WT and KO ESCs.
E Immunoblot of whole cell lysates from WT and H3.3 KO ESCs showing expression levels of NANOG, KLF4, OCT4 and SOX2. Total histone H3 was used as a loading control.

Fig. S11. Chromatin Landsacpe Is Dysregulated with H3.3 Loss

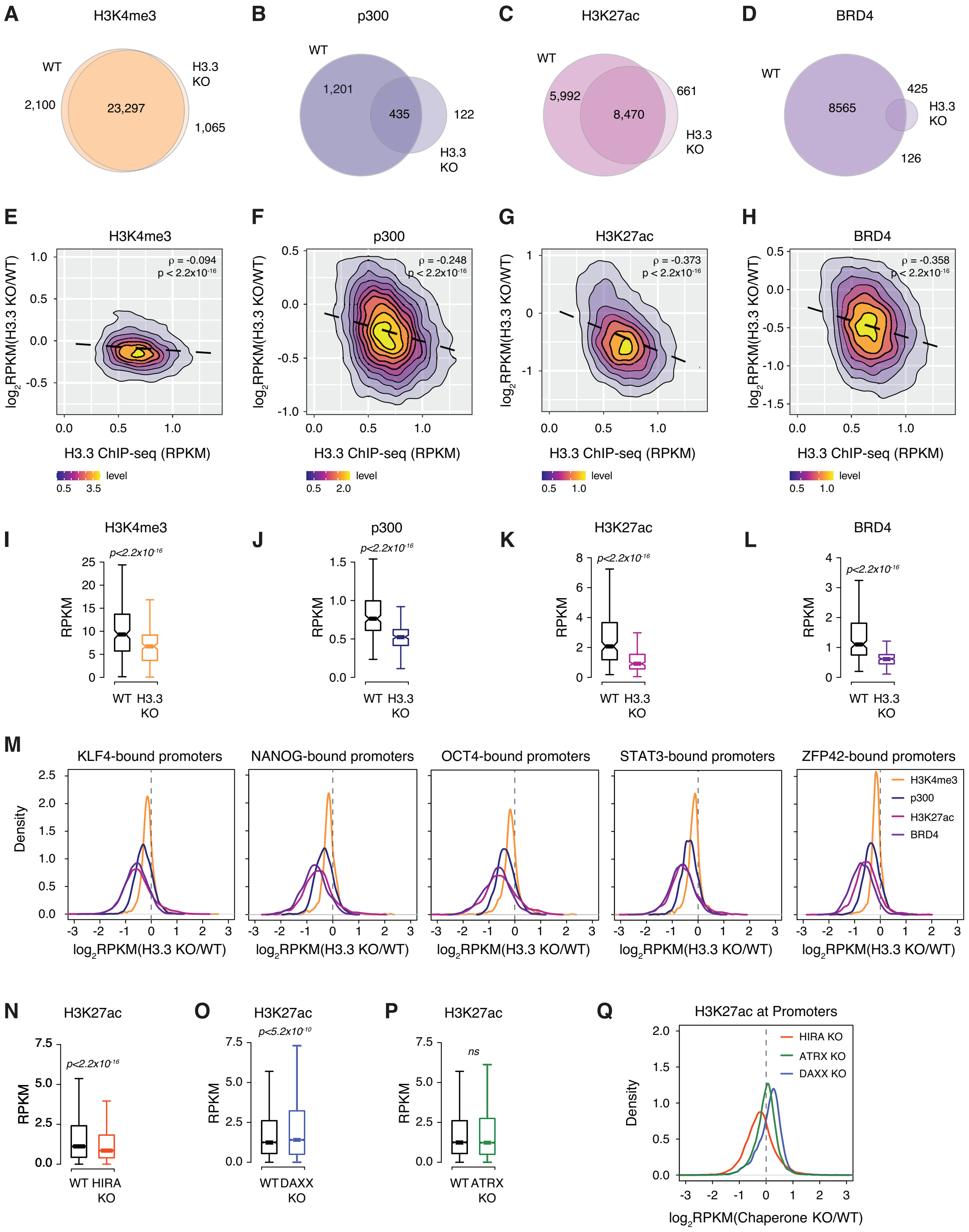

Fig. S11. Related to Figure 3. **Chromatin landscape is dysregulated with H3.3 loss.**

A-D Venn diagram showing overlap between promoters enriched with **(A)** H3K4me3, **(B)** p300, **(C)** H3K27ac, and **(D)** BRD4 in WT and H3.3 KO ESCs.
E-H Correlation plot between differential **(E)** H3K4me3, **(F)** p300, **(G)** H3K27ac, and **(H)** BRD4 enrichment in H3.3 KO compared to WT ESCs and H3.3 enrichment at active promoters (n = 12,903) in WT ESCs.
I-L Boxplots showing **(I)** H3K4me3, **(J)** p300, **(K)** H3K27ac, and **(L)** BRD4 enrichment at "H3.3-dependent" promoters in WT and H3.3 KO ESCs (n = 265).
M Ratio (log2) of H3K4me3, p300, H3K27ac, and BRD4 enrichment at promoters bound by the indicated TF in WT and H3.3 KO ESCs. x axis values <0 indicate reduced enrichment in the absence of H3.3.
N-P Boxplots showing H3K27ac enrichment at active promoters in WT and **(N)** HIRA KO, **(O)** DAXX KO, and **(P)** ATRX KO ESCS (n = 12,903). For all box plots, the bottom and top of the boxes correspond to the 25th and 75th percentiles, and the internal band is the 50th percentile (median). The plot whiskers correspond to 1.5x interquartile range and outliers are excluded. P-values determined by Wilcoxon rank sum two-side test.
Q Ratio (log2) of H3K27ac enrichment at active promoters (n = 12,903) in WT, HIRA KO, ATRX KO, and DAXX KO ESCs. x axis values <0 indicate reduced enrichment in the absence of chaperone.

Fig. S12. Loss of H3.3 alters RNAPII activty

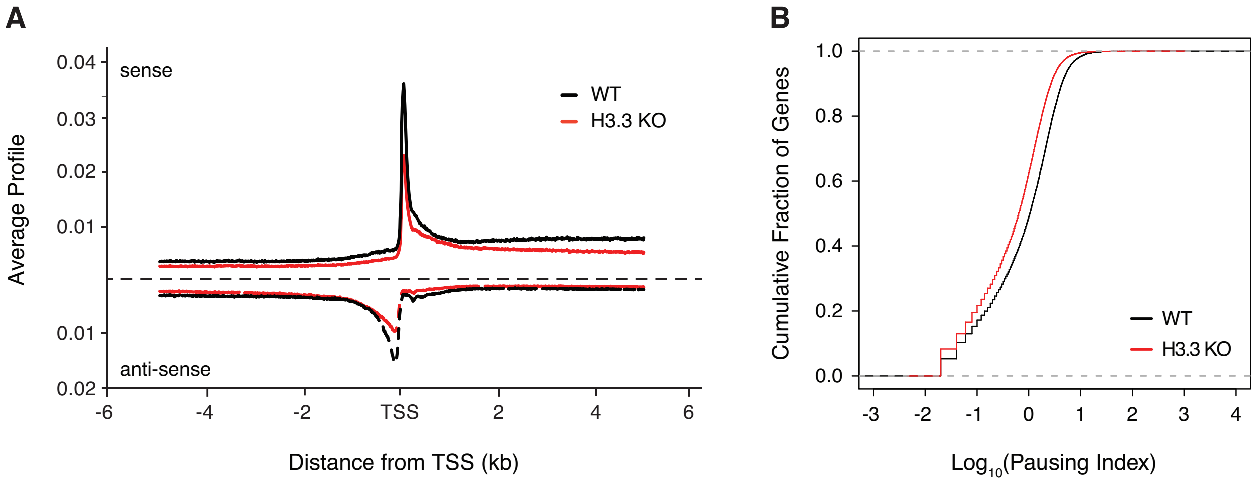

Fig. S12. Related to Figure 4. **Loss of H3.3 alters RNAPII activity.**

A Average profile of GRO-seq signal near the TSS of active promoters (n=12,903) in WT and H3.3 KO ESCs.

B Cumulative distribution of Pause Index defined as ratio between gene body (+300 bp to +10 kb of TSS) and promoter proximal (-300 bp to +300 bp of TSS) GRO-seq RNAPII density in WT and H3.3 KO ESCs.

Fig. S13. Transcriptional changes associated with loss of H3.3 in ESCs and during differentiation.

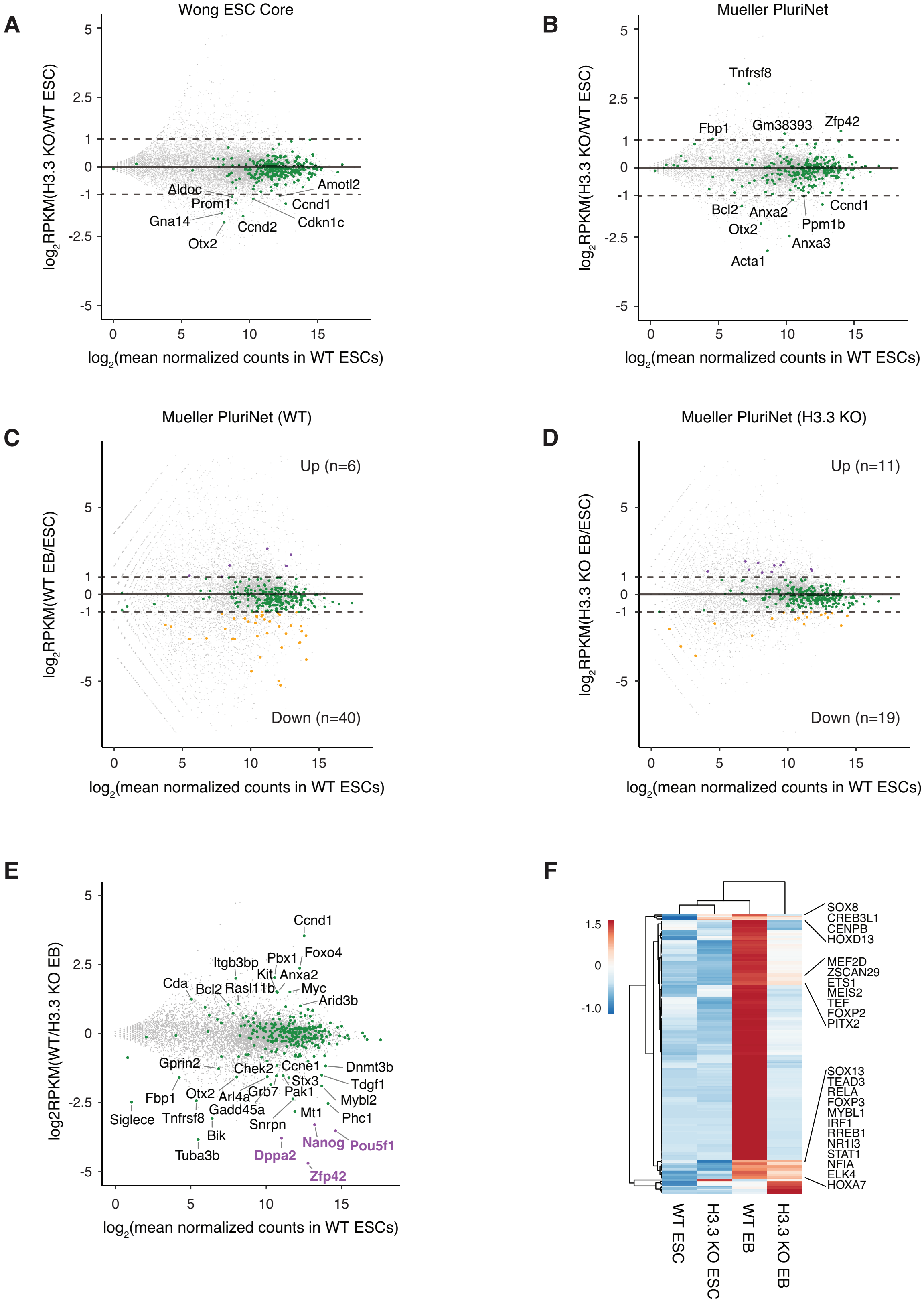

Fig. S13. Related to Figure 5. **Transcriptional changes associated with loss of H3.3 in ESCs and during differentiation.**

A-B MA plot of gene expression in WT and H3.3 KO ESCs. Members of **(A)** the core ESC-like gene module or **(B)** PluriNet are shown in green. Mean expression across compared samples is represented on the x-axis and differential expression between WT and H3.3 KO ESCs is represented on the y-axis.

C-D MA plot of gene expression in **(C)** WT and **(D)** H3.3 KO ESCs and EBs. Mean expression across compared samples is represented on the x-axis and differential expression between ESCs and EBs is represented on the y-axis. Members of the PluriNet gene set are shown in green (<2-fold change), purple (>2-fold increase in EBs) or orange (>-2-fold decrease in EBs).

E MA plot of gene expression in WT and H3.3 KO EBs. Mean expression from ESCs is represented on the x-axis and differential expression between WT and H3.3 KO EBs is represented on the y-axis. Differentially expressed genes are labeled in green and representative pluripotency genes are labeled in purple.

F Heatmap of transcription factor expression in WT and H3.3 KO ESCs and EBs. Represented TFs were upregulated ($\log_2$ fold-change > 0.5, p-value < 0.05) from WT ESCs to WT EBs. Highlighted transcription factors were not statistically different between WT and H3.3 KO EBs.

Fig. S14. Uncropped blots from Figure 6

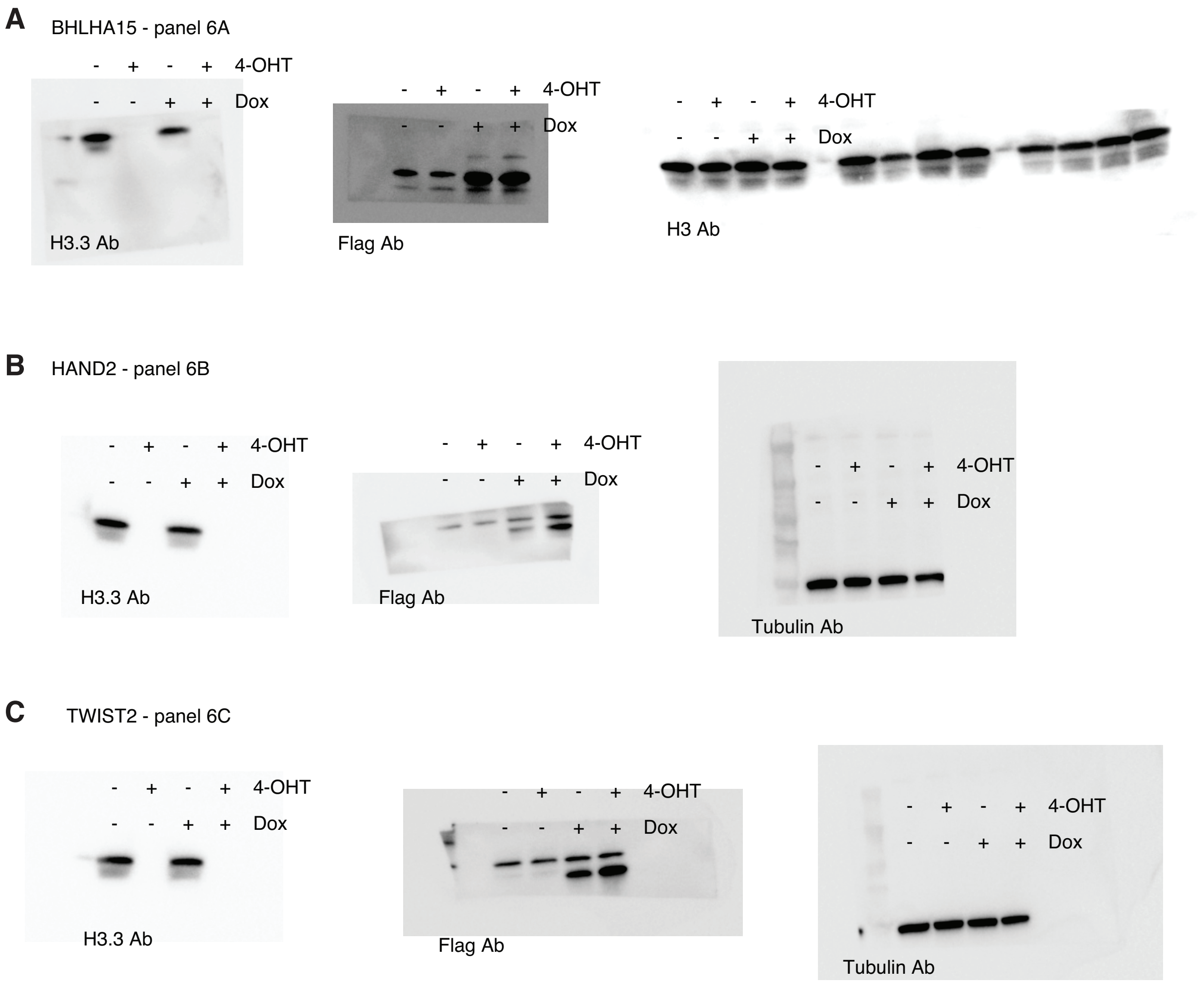

Fig. S14. Related to Figure 6. **Uncropped blots from Figure 6.**
A-C Immunoblot of whole cell lysates from *H3f3a*$^{fl/fl}$*;H3f3b*$^{fl/fl}$*;GtROSA26*$^{CRE\text{-}ERT2}$ ESCs treated with either EtOH or 2 μM 4-Hydroxytamoxifen for 48 hrs followed by treatment with either DMSO or 0.5 μg/ml doxycycline for 48 hrs showing expression levels of H3.3 and Flag-tagged **(A)** BHLHA15, **(B)** HAND2, or **(C)** TWIST2. * non-specific band ~30 kDa observed with Flag antibody in all lysates. Total histone H3 or β-tubulin are used as loading controls.

Fig. S15. Exogenously expressed TFs bind at both open and closed chromatin in WT ESCs

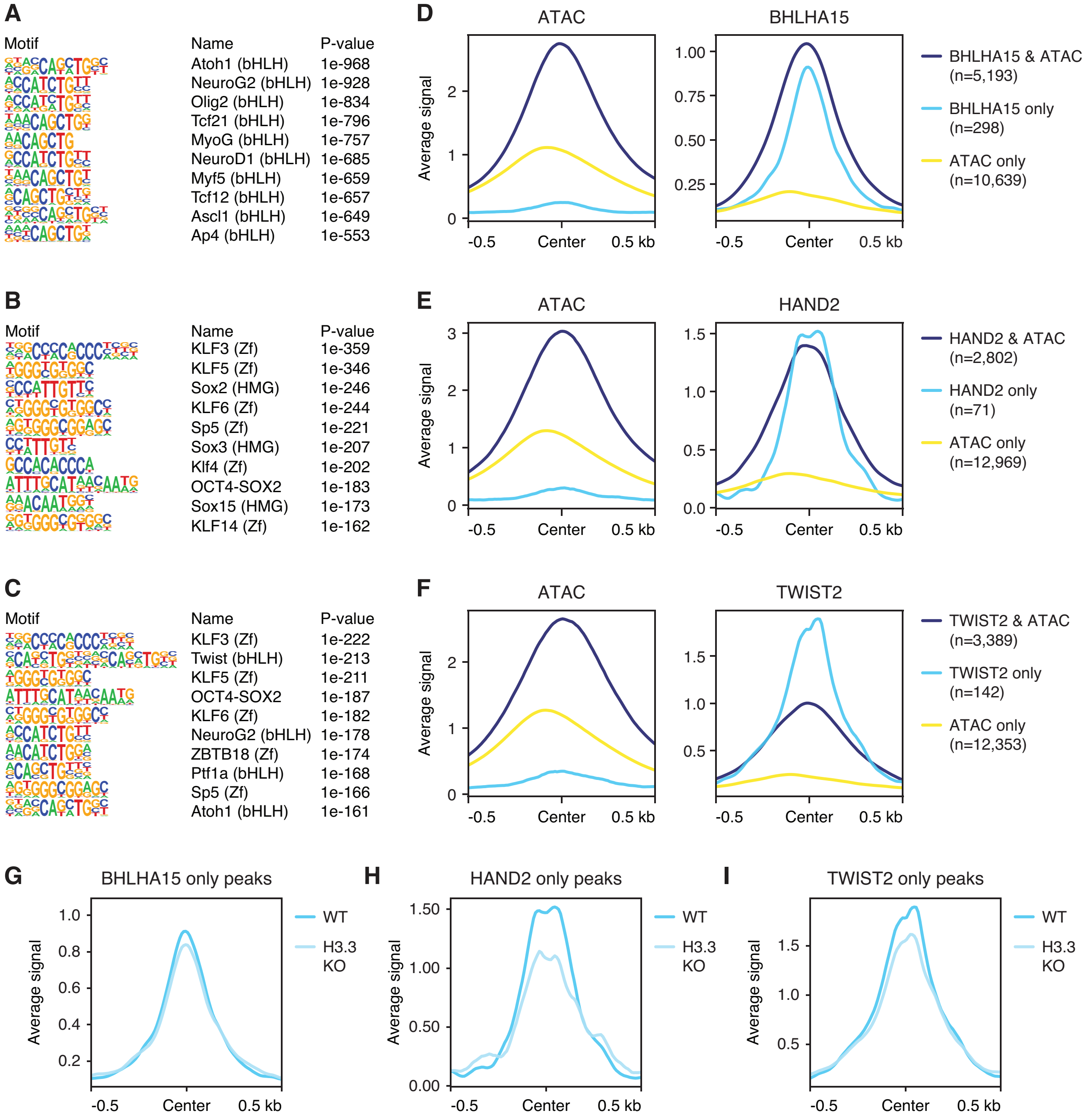

Fig. S15. Related to Figure 6. **Exogenously expressed TFs bind at both open and closed chromatin in WT ESCs.**

A-C Motifs enriched at regions enriched with **(A)** BHLHA15-Flag, **(B)** HAND2-Flag, or **(C)** TWIST2-Flag in WT ESCs. Known motif analysis was performed using HOMER.

D Average profile of ATAC-seq (left) and BHLHA15 CUT&Tag (right) at regions enriched for both ATAC and BHLHA15 binding (n=5,193), BHLHA15 only (n=298), or ATAC only (n=10,639) in WT ESCs.

E Average profile of ATAC-seq (left) and HAND2 CUT&Tag (right) at regions enriched for both ATAC and HAND2 binding (n=2,802), HAND2 only (n=71), or ATAC only (n=12,969) in WT ESCs.
F Average profile of ATAC-seq (left) and TWIST2 CUT&Tag (right) at regions enriched for both ATAC and TWIST2 binding (3,389), TWIST2 only (n=142), or ATAC only (n=12,353) in WT ESCs.
G-I Average profile of TF CUT&Tag at TF only enriched regions in WT and H3.3 KO ESCs for **(G)** BHLHA15, **(H)** HAND2, and **(I)** TWIST2.
