# Supplementary material for: H3.3 contributes to chromatin accessibility and transcription factor binding at promoter-proximal regulatory elements in embryonic stem cells
Source: Genome Biol. 2023 Feb 13;24:25. doi: 10.1186/s13059-023-02867-3 (PMC9926682; doi:10.1186/s13059-023-02867-3)
Supplement: Supplementary file 6 — Additional file 6. Review history. [file 13059_2023_2867_MOESM6_ESM.docx]

**1^st^ round**

**Reviewer 1**

In this manuscript, Tafessu et al study how histone variant H3.3 contributes to the establishment of chromatin state and transcription factor binding at gene promoters (and consequently transcription regulation) in self-renewing and differentiating mouse embryonic stem cells (ESCs).

First, the authors used ATAC-Seq to probe chromatin accessibility in WT and H3.3 KO ESCs and find a "slight" (~10-15%) decrease in chromatin accessibility at active gene promoters, which they show correlates, albiet weakly (R = 0.194), with H3.3 enrichment in WT ESCs. Genome-wide, they identified 265 regions (promoters?) that are less accessible in H3.3 KO ESCs and 70 regions that are more accessible in H3.3 KO ESCs. They were able to reproduce these observations in ESCs lacking HIRA, the H3.3 chaperone required for H3.3 enrichment at promoters of active and repressed genes, but not in ESCs lacking ATRX or DAXX, H3.3-associated proteins specifically required for enrichment of H3.3 at repeat regions such as telomeres. Based on these observations, they go on to hypothesize that this reduced promoter accessibility in H3.3 KO ESCs should be accompanied by reduced transcription factor (TF) binding.

Next, to test this, they utilize a recently published bioinformatics method called TOBIAS [??] to probe TF binding site (BS) footprints, regions within accessible chromatin (ATAC-Seq) protected by TF binding. Through comparative TF footprinting analysis of 395 expressed TFs (with known binding motifs) in WT and H3.3 KO ESCs, they find evidence for reduced binding (~10-20%) of nearly all expressed TFs in H3.3 KO cells. Given that H3.3 KO does not affect expression of TFs, they conclude that the loss of H3.3 affects binding of TFs to promoters. They go on to show that besides the inferred decrease in TF binding, loss of H3.3 also results in reduced promoter enrichment of H3K4me3, p300, H3K27ac, and BRD4 (modifications/cofactors generally associated with transcriptional activity) and reduced levels of nascent (GRO-Seq) but not steady-state (RNA-Seq) transcription.

Finally, through comparative TF footprinting analysis of ATAC-Seq data in WT and H3.3 KO embryoid bodies (EBs), they show that, unlike in WT EBs, pluripotency-associated TFs tend to remain bound to their motifs in H3.3 KO EBs, consistent with their earlier study showing that H3.3 is required for proper differentiation of ESCs to EBs. Based on all of their findings, they conclude that although H3.3-mediated TF binding may not be required to maintain gene expression programs controlling ESCs, H3.3 deposition is essential for establishing a chromatin state that promotes binding of differentiation-associated TFs.

1. The authors focus their investigation on gene promoters and use "promoters" throughout the manuscript. It was never clear whether by "promoters" they are referring to "all gene promoters" or only those gene promoters/regions that exhibit differential accessibility in H3.3 KO cells (H3.3-dependent; n = 265). They also sometimes use "active promoters"; are these "H3.3 dependent" and transcriptionally active? Are there H3.3-dependent and transcriptionally silent promoters? It was a challenge trying to pin down the set of promoters that is being investigated for various analyses. Please clarify. And make sure to include the number of promoters used for every analysis (e.g., n= 265) within figures/legends.

2. To make sure that the observed effects are due to direct effects of H3.3 KO, it would make sense for the authors to consider only those promoters enriched for H3.3 in WT ESCs and whose chromatin accessibility decreases in H3.3 KO ESCs (n = 265).

3. Based on their previous paper [29], we know that H3.3 promotes PRC2 binding at the promoters of transcriptionally silent genes. Please clarify whether these promoters are included/excluded in their analysis? And, how is H3.3's function at active promoters (contribution to chromatin accessibility) reported in this paper different from H3.3's function at transcriptionally silent PRC2/bivalent sites reported in their previous paper?

4. The authors spend a bulk (~80%) of the paper presenting results showing the effects of H3.3 KO in mouse ESCs: the loss of H3.3 minimally affects (reduces) chromatin accessibility, TF binding, and enrichment for chromatin marks associated with transcription activation, and nascent transcription. Yet, these observed changes (10-20% reduction) have no impact on steady-state transcript levels, raising the question as to whether the observed changes, though statistically significant, are biologically meaningful.

5. In the last part of the paper, they argue that H3.3 is essential for establishing new TF binding during differentiation of ESCs to embryoid bodies (EBs). By this, do they mean that H3.3 is required at promoters that are not active in ESCs, but get activated during differentiation? Or do they mean that H3.3 is required at promoters that are active in ESCs in order for these genes to properly silenced during differentiation?

6. In the previous paper [29], they reported "no delay in downregulation of pluripotency factors (data not shown)" in H3.3 KO EBs vs WT EBs. In this paper, they conclude, based on TOBIAS footprinting analysis, that ESC TFs remain bound to their motifs in H3.3 KO EBs, unlike in WT EBs. Does this mean ESC TFs are expressed but are unable to access their binding sites in KO EBs? If so, please consider presenting ChIP-Seq of a handful of key ESC TFs showing that their binding is reduced in KO ESCs. At the least ChIP-qPCR analysis of a handful of TFBSs overlapping H3.3 peaks showing reduced binding in KO cells would suffice; as negative controls, include a set of TFBSs not overlapping H3.3 peaks (and/or not showing reduced binding).

7. Please include gene expression and WB analysis of key ESC factors in WT and KO EBs.

8. In an earlier work from the Allis lab (in which the senior author of this paper is a co-author), it was shown that Hira is not essential for the H3.3 enrichment at TFBSs (e.g., Oct4, Sox2, Nanog, Smad1, Stat3) in ESCs. If Hira is not essential for H3.3 enrichment at TFBSs, please explain why the results from the TOBIAS footprinting analysis in Hira KO ESCs are the same as those in H3.3 KO ESCs (Fig. 2A and S4A)? Specifically, if Hira is not essential for H3.3 enrichment at TFBSs, shouldn't we expect to see no differences in TFBS footprinting in Hira KO vs WT ESCs? Please clarify.

9. Results from the TFBS footprinting analysis suggest reduced binding of nearly all 395 expressed TFs analyzed, which makes one question whether results from this analysis are reliable. To strengthen this analysis, the authors should consider ChIP-Seq or ChIP-qPCR analysis of a handful of TFBSs overlapping H3.3 peaks showing reduced binding in KO cells; as negative controls, include a set of TFBSs not overlapping H3.3 peaks (and/or not showing reduced binding).

10. Base on Fig. 3 data, the authors argue that H3K4me3 at active promoters has "little reliance" on H3.3 whereas p300, H3K27ac, and BRD4 exhibit "reduced enrichment" although the p-value for the reduced signal in all four cases is <2.2x10E-6. Not sure if this conclusion is justifiable. Please clarify. Also include density profiles for H3K4me3, p300, H3K27ac, and BRD4 (as in Fig. 1C and 2B).

11. Fig. 1E: please also include box plots for all promoters (Fig. 1C).

12. Fig. S2B and related text: please consider adding a Venn diagram showing the overlap between H3.3 dependent promoters, HIRA-dependent promoters, and ATRX/DAXX-dependent promoters.

Minor points:

13. Fig. S3: panels B, D, F, H missing p-values.

14. Line 170-172, "Loss of TF binding …" should be "Reduced/decreased TF binding". Similarly, "global loss of TF expression" should be "decrease in TF expression"

**Reviewer 2**

The functional role of histone H3.3 is not fully understood, and its pleotropic effects have been revealed in various types of genomic regions, possibly due to its role as a fundamental structural element of chromatin templates. In this manuscript, Tafessu and colleagues describe a series of phenotypes associated with TSS regions in H3.3 KO mESCs. Using epigenome profiling techniques including ATAC-seq and ChIP-seq, the authors show that in H3.3 KO mESCs, the average of chromatin accessibility in TSS regions is mildly reduced, along with a concomitant decrease of histone modifications and transcriptional co-activators all of which are associated with active transcription. By performing GRO-seq, the authors revealed that nascent RNA levels around TSS region were also reduced in H3.3 KO mESCs, further suggesting a role for H3.3 in active gene transcription. Since mESCs lacking H3.3 showed minor defects in gene expression as measured by RNA-seq, the authors then used ATAC-seq to show that TF bindings is impaired during mESC differentiation in H3.3 KO EBs. Finally, they propose a function of H3.3 in facilitating TF binding at newly active promoters.

Overall, the conclusions are not convincing enough, especially the TF binding part. The differences described for the promoter regions between WT and H3.3 KO are relatively mild and most analyses are quite crude. Moreover, I am concerned about the observation of ATAC-seq signal loss at the TSSs after losing H3.3 and its interpretation. I suspect the minor alterations observed in the study is caused by normalization.

Major Point:
1. The authors revealed a trend of decrease in ATAC-seq signal at the TSS regions, yet no such trend at enhancer regions. Enhancers are also enriched for TFs and H3.3, it is hard to reconcile their results with their model.

2. The authors suggest that TF binding at the TSS is also compromised due to loss of H3.3. It is quite counterintuitive that chromatin becomes less accessible, when both TFs and histones are locally reduced.

3. The authors should analyze the ATAC-seq signal across the genome, to address whether their observed accessibility loss is an indirect consequence of normalization. This is particularly important because an increase of ATAC-seq signal at repetitive elements has been reported before (e.g., Carmen Navarro et al., 2020). During data processing, sequencing reads were normalized to the same depth for comparison. Is the small differences at the TSS regions a secondary effect of data normalization?

4. The authors claim that the ATAC-seq peaks decrease at TSS regions. However, as shown in Fig. S2B, both increase and decrease of ATAC-seq signal can be detected at different TSSs. What is the difference between the two groups with different response?

5. By analyzing ATAC-seq data at the EB stages, the authors suggest that H3.3 KO may affect TF binding at novel binding sites during differentiation. What are the gene expression levels of differentiation-specific TFs in H3.3 KO EBs? If these TFs also show differential expression level, I cannot agree with the authors, instead it can simply reflect a global defect in differentiation. For example, the authors attempted to claim that H3.3 deposition is essential for establishing new TF binding patterns during differentiation, as lineage-specific TFs showed greater binding in WT EBs (Fig 5B). However, HAND2, GATA3, GATA2, TWIST2 was not even activated in H3.3-loss EBs, evidenced by RNA-seq data they used. Similarly, pluripotency-maintaining TFs (KLF5, KLF4, NANOG, POU5F1, ZFP43 et. al) were not silenced in the absence of H3.3 during EB differentiation. Therefore, the expression of corresponding TFs, but their chromatin binding, is critical for differentiation.

6. The authors identified only 265 regions with lower accessibility in H3.3-depleted mESCs (Fig S2A). However, it seems that p300 and BRD4 recruitment at all promoters was disturbed (Fig 3, n=12,903), and the same is true for GRO-seq signal at promoters of expressed genes (Fig 4A). Any reasonable explanation?

7. It is surprising to see that only 1636 promoters were enriched with p300 (Fig S6B).

Minor Point
1. line 120-122. As shown in Fig S2B, the number of differential ATAC-seq peaks at enhancer regions are in fact more than those at TSS regions. It is not appropriate to claim that there is NO change at the enhancer regions.
2. CTCFL is not expressed in mouse ES cells. It does not make sense to include its binding motif in Fig 2C.
3. The total number of promoters and enhancers should be indicated in the figures.

**Reviewer 3**

The histone H3 variant, H3.3, is essential for development, as knockout of H3.3 in mice is lethal due to severe developmental defects. Hence, understanding the detailed mechanism of how H3.3 regulates transcriptional programs in development is of great interest and significance.

The manuscript by Tafessu et al., specifically focused on the chromatin accessibility of promoter-proximal regulatory regions upon loss of H3.3, then characterized the related chromatin status in terms of H3K27ac, its writer (p300) and reader (BRD4) as well as RNA ploymerase, finally defined footprintings of ESC- and lineage-specific TFs accounting for the diminished capacity of H3.3 KO mESC to differentiate. This is an extended study from their previous work (Martire et al., Nat Genet, 2019) where they reported that the H3.3-regulated enhancers, together with H3K27ac and p300 are involved during mESC differentiation. Compared to their previous study, they provided new proofs from the perspective of H3.3-bound promoters to explain the compromised ability of H3.3 KO mESC to differentiate. The new findings in the present study include: i) the H3.3-bound promoter regions are characterised; ii) they integrated RBD4 and RNA polymerase into the picture; iii) the footprintings of ESC- and lineage-specific TFs are defined. However, this study, in its current form, did not provide very convincing data, due to the lack of the validation experiments and the weak/very mild correlation between the high-throughput sequencing data.
The following are my concerns.

Major concerns:

1. In Line 131, the authors claimed that "In agreement with previous studies, we find that H3.3 enrichment at active regulatory elements is correlated with accessibility", as shown in Fig. 1A and Fig. S1A. However, the correlation coefficient (ρ) showed only 0.463 and 0.2 for promoters and enhancers, respectively, indicating mild or no correlations. Also, in Figure D, the correlation (ρ=-0.194) seems too weak to support the statement "Globally, we find that reduced ATAC-seq signal at promoters in H3.3 KO ESCs is correlated with H3.3 enrichment in WT ESCs (Line 125)". The same for Figure 4B (ρ=0.38) as well as Fig. S6 F (ρ=-0.248), G (ρ=-0.373) and H (ρ=-0.358). The authors should avoid being over-confident in their conclusions.
2. Concerning the H3.3 ChIP-seq data (Figure 1A and 1D), could the authors show the global heatmap and some representative examples? Also, did the authors perform ChIP-seq under H3.3 KO condition, together using IgG antibody as negative controls in order to obtain H3.3 peaks of high confidence?
3. In Fig. 1B, C and Fig. S1B, the authors observe a slight but significant decrease in chromatin accessibility at promoters but not distal regulatory elements (enhancers) in the absence of H3.3. However, in Supplementary Figure 2A, it shows that the number of differentially accessible enhancers is more than that of promoters in H3.3 KO compared to WT ESCs, indicating that the accessibility in both enhancers and promoters is changed. Could the authors explain this?
4. As shown in Figure 4 A and B, the binding of key TFs e.g. POU5F1, NANOG, SOX2 are differentially changed, together with the pol II engagement at promoters upon depletion of H3.3, although the global transcription remains undisturbed in ESC stage. Also in Figure 2B, the reduction in promoter binding by e.g. OCT4 and NANOG seems very minor. Therefore, some top candidates genes/well-known OCT4/NANOG target even their own promoters should be assessed by e.g ChIP-qPCR, given that POU5F1, NANOG, SOX2 forms an interconnected autoregulatory circuit, by targeting both their own promoters and those of each other. Similar experiments should be applied to the EBs in terms of lineage-specific TFs.
5. In Supplementary Figure 7, the authors showed the transcriptional changes with loss of H3.3 in ESCs and during differentiation. However, the comparison between H3.3KO EBs and WT EBs is missing.
6. Concerning the differentiation strategy, the authors employed the undirected differentiation (Line 35 and 36) to evaluate the effect of loss of H3.3. In this way, the heterogeneity in the cell population might make the data interpretation difficult and inaccurate. Perhaps directed differentiation would be better. The authors are encourage to discuss this in the discussion section.

Minor concerns:

1. In Figure 3A, for the first panel, the Heatmaps of ATAC-seq in both WT and H3.3KO should be shown for a better comparison.
2. In Methods part, the mES cell line (Line 366) should be specified.
3. Regarding the differentiation protocol, did the authors adopt it from a literature or not? How efficient is the protocol? Did they analyze any marker genes?
4. In Supplementary Figure 3, the exact P values should be shown either in figure or in figure legend.

**Reviewer 4**

Summary of Results: Histone variant H3.3 has been established to be present at dynamically regulated portions of chromatin such as promoters, gene bodies, and enhancers. However, the exact function of H3.3 in maintaining the proper accessibility and function of these regions is not fully understood. In this study, the authors identify promotor dysregulation in the absence of H3.3 in mouse embryonic stem cells. This decreased accessibility corresponded to decreased TF foot printing (reduced accessibility to chromatin binding by TFs?) & dysregulation of the chromatin landscape in these cells (secondary to reduced TF binding?), without dramatically changing transcriptional output (heterogeneity and selection of ESC with reduced transcriptional defects?). However, the authors show decreased RNAPII engagement in the TSS of expressed genes, which implies that proper TF binding and RNAPII engagement requires promotor proximal H3.3. What the mechanism is that links H3.3 deficiency to reduced TF binding and RNAPII activity is unclear and yet may be readily illuminated with minimal additional effort, strengthening the paper and its utility to the field.

The manuscript is scholarly and well written and in general the conclusions derived from the data are sound. It is an important contribution to the H3.3 field and an understanding of this important histone variant in transcription. However, it is critical for interpretation and mechanistic understanding of the phenotypes described for the authors to perform experiments, analysis, and provided discussion of how nucleosome occupancy is changing following H3.3 KO (see point 1, below).

Major Comments:

1) Is H3.1/2 is localized to regions normally occupied by H3.3 in the H3.3 KO cells? ES cells are rapidly cycling and S-phase expressed H3.1/2 could be occupying the nucleosomes at these regions. Thus, since H3.1/2 is generally considered to me more stable in a nucleosome with H2A.Z than H3.3, could this explain the decreased accessibility, reduced TF binding, with transcriptional changes. To address this H3.3 and a H3.1/2 ChIPs (and/or pan H3) should be performed in WT/KO cells. Additionally, H2A.Z ChIPs would be interesting but are not necessary for interpretation of the main point of nucleosome occupancy, but rather go beyond to address composition and classes of promoters that may be regulated by H2A.Z/H3.3 nucleosomes.

2) The data that reveal regulation of H3.3 deposition by HIRA complex vs ATRX/DAXX are interesting. However, the conclusions would be strengthened by ChIP for H3.3 in chaperone knock out experiments. Does H3.3 deposition increase in DAXX KO at some loci? To what extent does H3.3 deposition decrease in HIRA KO? HIRA complex could also have function in local H3.3 turnover and activities on H3.3 bound chromatin independent and beyond function in H3.3 deposition.

3) The GRO-seq experiment in Figure 4 provides an interesting insights but the authors don't follow up (experimentally or with sufficient discussion of possible interpretations) on any potential mechanism that is causing the reduced engagements of RNAPII with promoters in the absence of H3.3. While "TF footprint" analysis suggests reduced TF binding, the computational approaches employed are not strictly footprinting analysis, rather motif-proximal/associated accessibility. To infer chromatin binding by TF more directly the authors should employ other tools dedicated for this kind of analysis, for example HINT: https://doi.org/10.1186/s13059-019-1642-2. Such analysis could improve these interpretations. However, moving beyond TF binding and links to RNAPII recruitment and activity as interpreted by GRO-seq, the authors only provide superficial GRO-seq data visualization and analysis. Can they show some example tracks of these data with characteristic reductions in 5' GRO-seq signal in H3.3 KO cells? Can they interpret ratio of 5' to 3' GROseq to make inferences such as that H3.3 KO is reducing RNAPII initiation but not reducing elongation rates, etc?

Minor Comments:

1) What about ERVs in ESC? The last author previously published on H3.3 enrichment at ERVs and recent studies have further highlighted that H3.3 may regulate ERV expression, perhaps linked to bivalent chromatin (https://www.nature.com/articles/s41556-021-00795-7). Do the authors relate these results to the ERV de-repression and nucleosome depletion/accessibility at these regions.

2) The authors interpret their results to say that H3.3 is necessary for nucleosome displacement to occur. But the paradox of deleting H3.3 potentially leading to nucleosome depletion is not addressed. These ideas—nucleosome depletion vs replacement with H3.1/2 nucleosomes should be discussed and presented clearly for readers to accurately interpret the results presented.

3) The authors don't mention all the antibodies they used for ChIP-seq (i.e. anti-H3K27ac) or show any antibody validation data (for example equivalent binding of K27ac on H3.3 vs H3.1/2 is critical—if data is available, great, if not, it is critical for the specific reagent to be specified and any available specificity data presented).

4) The subfigures in Figure 3 seem somewhat redundant as the heatmap, boxplot, and density plot are all conveying same message of decreases these active histone marks & their cofactors at promoters in H3.3 KO ES cells. What about the promoters (and associated genes) where this effect is the most drastic? Is this affect the same genome wide or a similar trend to the change in accessibility where the promoters with the most H3.3 in WT cells have the greatest decrease in H3K27ac, p300, etc.?

5) Why aren't HIRA, ATRX, & DAXX KO ES cells used in the differentiation experiment in Figure 5 as a comparison to H3.3 KO (as they are for much of the rest of the study)?

**Authors’ response**

Based on reviewer suggestions, we have substantially revised our manuscript, outlined in more detail below. This includes major experimental and computational revision. These include:
1) TF CUT&Tag experiments to validate our ATAC-seq data
2) A more nuanced analysis of chromatin accessibility at enhancers and promoters
3) TF CUT&Tag experiment to address the effect of H3.3 on de novo TF binding
4) More balanced discussion of our data in context of the current literature
Overall, we feel that our manuscript has greatly improved through the review process. Please find our response to specific comments below.
Reviewer Comments
Reviewer #1: In this manuscript, Tafessu et al study how histone variant H3.3 contributes to the establishment of chromatin state and transcription factor binding at gene promoters (and consequently transcription regulation) in self-renewing and differentiating mouse embryonic stem cells (ESCs).
First, the authors used ATAC-Seq to probe chromatin accessibility in WT and H3.3 KO ESCs and find a "slight" (~10-15%) decrease in chromatin accessibility at active gene promoters, which they show correlates, albiet weakly (R = 0.194), with H3.3 enrichment in WT ESCs. Genome- wide, they identified 265 regions (promoters?) that are less accessible in H3.3 KO ESCs and 70 regions that are more accessible in H3.3 KO ESCs. They were able to reproduce these observations in ESCs lacking HIRA, the H3.3 chaperone required for H3.3 enrichment at promoters of active and repressed genes, but not in ESCs lacking ATRX or DAXX, H3.3- associated proteins specifically required for enrichment of H3.3 at repeat regions such as telomeres. Based on these observations, they go on to hypothesize that this reduced promoter accessibility in H3.3 KO ESCs should be accompanied by reduced transcription factor (TF) binding.
Next, to test this, they utilize a recently published bioinformatics method called TOBIAS [??] to probe TF binding site (BS) footprints, regions within accessible chromatin (ATAC-Seq) protected by TF binding. Through comparative TF footprinting analysis of 395 expressed TFs (with known binding motifs) in WT and H3.3 KO ESCs, they find evidence for reduced binding (~10-20%) of nearly all expressed TFs in H3.3 KO cells. Given that H3.3 KO does not affect expression of TFs, they conclude that the loss of H3.3 affects binding of TFs to promoters. They go on to show that besides the inferred decrease in TF binding, loss of H3.3 also results in reduced promoter enrichment of H3K4me3, p300, H3K27ac, and BRD4 (modifications/cofactors generally associated with transcriptional activity) and reduced levels of nascent (GRO-Seq) but not steady-state (RNA-Seq) transcription.
Finally, through comparative TF footprinting analysis of ATAC-Seq data in WT and H3.3 KO embryoid bodies (EBs), they show that, unlike in WT EBs, pluripotency-associated TFs tend to remain bound to their motifs in H3.3 KO EBs, consistent with their earlier study showing that H3.3 is required for proper differentiation of ESCs to EBs. Based on all of their findings, they conclude that although H3.3-mediated TF binding may not be required to maintain gene expression programs controlling ESCs, H3.3 deposition is essential for establishing a chromatin state that promotes binding of differentiation-associated TFs

1.The authors focus their investigation on gene promoters and use "promoters" throughout the manuscript. It was never clear whether by "promoters" they are referring to "all gene promoters" or only those gene promoters/regions that exhibit differential accessibility in H3.3 KO cells (H3.3-dependent; n = 265). They also sometimes use "active promoters"; are these "H3.3 dependent" and transcriptionally active? Are there H3.3-dependent and transcriptionally silent promoters? It was a challenge trying to pin down the set of promoters that is being investigated for various analyses. Please clarify. And make sure to include the number of promoters used for every analysis (e.g., n= 265) within figures/legends.
We have clarified the set of promoters used throughout the manuscript, both in the text as well as in the figure legends. The majority of the analyses focus on transcriptionally active promoters, determined as baseMean >20 by RNA-seq across WT and H3.3 KO ESC data sets (n = 12,903). The bed file of computationally defined “H3.3-depdendent” promoters (n= 265) is used only in Supplemental Figure 5 and Supplemental Figure 11. Based on new analysis completed in revision (see Supplemental Figure 5E-I), we think that these promoters represent highly expressed genes with chromatin signatures of activity in WT ESCs. Our general understanding is that any promoter with H3.3 deposition will likely be experiencing some level of RNAPII activity, and that the level of H3.3 enrichment is related to the level of transcription.

2. To make sure that the observed effects are due to direct effects of H3.3 KO, it would make sense for the authors to consider only those promoters enriched for H3.3 in WT ESCs and whose chromatin accessibility decreases in H3.3 KO ESCs (n = 265).
Given the genome-wide correlations that we observe, we prefer to keep the main analyses as presented. The “H3.3-dependent” group of promoters is a computational constraint rather than a biological annotation, with this group of promoters showing signatures of active chromatin and high transcription when compared to all expressed genes (see new Supplemental Figure 5E-I). It’s fun to speculate that this group of genes may be hypersensitive to changes in the quantity / composition of transcriptional co-activators recruited, the kind of cooperativity that has been proposed for the super-enhancer / condensate model of transcription. Indeed, we see that the effects of H3.3 loss on this group are more pronounced with respect to reduced enrichment of chromatin proteins and histone modification. These data are now included as a new Supplemental Figure 11I-L.

3. Based on their previous paper [29], we know that H3.3 promotes PRC2 binding at the promoters of transcriptionally silent genes. Please clarify whether these promoters are included/excluded in their analysis? And, how is H3.3's function at active promoters (contribution to chromatin accessibility) reported in this paper different from H3.3's function at transcriptionally silent PRC2/bivalent sites reported in their previous paper?
As stated by the reviewer, our previous work has shown that H3.3 is enriched at PRC2 targets, or “bivalent” loci in ESCs. These genes are defined by the presence of both H3K4me3 and H3K27me3 at their promoters. When we overlap a list of bivalent promoters with our list of active promoters, we find that ~73% of bivalent promoters were included in our list of active promoters (defined as >20 baseMean across WT and H3.3 KO ESC RNA-seq data). As expected, genes with bivalent promoters are lowly expressed compared to genes of all active promoters. Analysis of ATAC-seq data shows that bivalent promoters lose accessibility in H3.3 KO ESCs. This is also true in HIRA KO ESCs, but not ATRX or DAXX KO ESCs. We include these data for reviewer assessment below.
Our previous analysis (PMID 24074864, Banaszynski et al Cell 2013) showed that all promoters are subject to lower levels of de novo-synthesized histone deposition (via CATCH-IT analysis) and suggested that these promoters are more “closed” in the absence of H3.3 (via global H3 ChIP). We extend this last point by now including general H3 ChIP-seq analysis at active promoters, showing that we identify more H3 at these regions in two independent H3.3 KO ESC clones (included as a new Supplemental Figure S3K). Our current analyses using ATAC-seq support our previous conclusions with additional experimental evidence.

4. The authors spend a bulk (~80%) of the paper presenting results showing the effects of H3.3 KO in mouse ESCs: the loss of H3.3 minimally affects (reduces) chromatin accessibility, TF binding, and enrichment for chromatin marks associated with transcription activation, and nascent transcription. Yet, these observed changes (10-20% reduction) have no impact on steady-state transcript levels, raising the question as to whether the observed changes, though statistically significant, are biologically meaningful.
It is true that the observed changes in the pluripotent state are small, however the inability of H3.3 KO cells to establish new transcription programs when stimulated to differentiate is a notable phenotype. To this point, we add a new experiment demonstrating that H3.3 is needed for de novo expressed TFs to bind to chromatin, see responses below and new Figure 6. Our results, together with a growing body of literature, suggest that the effects of H3.3 on chromatin state may be more important during the initiation of new transcription programs rather than for the maintenance of ongoing transcription in cells.

5. In the last part of the paper, they argue that H3.3 is essential for establishing new TF binding during differentiation of ESCs to embryoid bodies (EBs). By this, do they mean that H3.3 is required at promoters that are not active in ESCs, but get activated during differentiation? Or do they mean that H3.3 is required at promoters that are active in ESCs in order for these genes to properly silenced during differentiation?
This is an excellent point and not something that we can distinguish from the experimental data provided in the initial submission. It is entirely possible that H3.3 is required both to set up the new transcription program and to turn the old one off.
To more directly address the requirement of H3.3 for TF binding, we have included a new experiment. Here, we express differentiation-specific TFs, in other words, TFs the cells have not experienced before, in both WT and H3.3 KO ESCs. Despite being expressed at similar levels, we find that occupancy on chromatin is lower in the H3.3 KOs. These data suggest that H3.3 does promote TF binding and are included as Figure 6. Please note that this experiment does not preclude a role for H3.3 in nucleosome turnover that may help to shut down the pluripotency transcription program.

6. In the previous paper [29], they reported "no delay in downregulation of pluripotency factors (data not shown)" in H3.3 KO EBs vs WT EBs. In this paper, they conclude, based on TOBIAS footprinting analysis, that ESC TFs remain bound to their motifs in H3.3 KO EBs, unlike in WT EBs. Does this mean ESC TFs are expressed but are unable to access their binding sites in KO EBs? If so, please consider presenting ChIP-Seq of a handful of key ESC TFs showing that their binding is reduced in KO ESCs. At the least ChIP-qPCR analysis of a handful of TFBSs overlapping H3.3 peaks showing reduced binding in KO cells would suffice; as negative controls, include a set of TFBSs not overlapping H3.3 peaks (and/or not showing reduced binding).
The referenced paper compared WT ESCs to ESCs treated with shRNA against the two H3.3 genes (H3.3 KD). As stated, we observed that these cells were able to exit pluripotency. Over the course of that study, we generated genetic H3.3 KO ESCs that were further characterized in PMID 31152160 (Martire et al, Nature Genetics 2019) where we report that H3.3 KO ESCs fail to downregulate pluripotency factors at both the transcript and protein level (See Supplementary Figure 7 of that publication). We attribute these distinct phenotypes to differences between KD and KO, with perhaps a relatively small amount of H3.3 being sufficient to allow transitions in cell state.
The TOBIAS analysis presented in the current submission are in line with the previous differentiation defects we reported for H3.3 KO ESCs. Our interpretation of these data is that ESC TFs, while less bound to their targets in H3.3 KO ESCs, remain more bound in H3.3 KO cells compared to WT when cells are subject to differentiation conditions.

The reviewer raises an excellent point that we should demonstrate genomic enrichment of key ESC TFs in WT and H3.3 KO ESCs. We now provide CUT&Tag analysis of two transcription factors, KLF5 and SOX2, showing that both of these TFs show reduced enrichment on chromatin in the absence of H3.3. These data are included as Figure 2E and Supplemental Figure 9E-F.

7. Please include gene expression and WB analysis of key ESC factors in WT and KO EBs. We now include RNA-seq analysis of WT vs H3.3 KO EBs, where we highlight a number of pluripotency (e.g., Oct4, Nanog, Rex1, Dppa2) factors that remain expressed in H3.3 KO EBs compared to WT EBs, where these factors are downregulated. This analysis is included as a new Supplemental Figure S12E.
Much of the data analyzed in this current study was previously submitted as GSE114551 and reported in PMID 31152160 (Martire et al, Nature Genetics 2019). Supplemental Figure 7 of this submission demonstrates the differentiation or lack thereof in WT and H3.3 KO EBs by RT-qPCR of pluripotency and lineage markers, RNA-seq analysis, and WB of the critical pluripotency factor Oct4. Collectively, these data demonstrate that H3.3 KO EBs maintain expression of critical regulators of pluripotency while WT EBs downregulate these factors in response to differentiation stimulus.

8. In an earlier work from the Allis lab (in which the senior author of this paper is a co-author), it was shown that Hira is not essential for the H3.3 enrichment at TFBSs (e.g., Oct4, Sox2, Nanog, Smad1, Stat3) in ESCs. If Hira is not essential for H3.3 enrichment at TFBSs, please explain why the results from the TOBIAS footprinting analysis in Hira KO ESCs are the same as those in H3.3 KO ESCs (Fig. 2A and S4A)? Specifically, if Hira is not essential for H3.3 enrichment at TFBSs, shouldn't we expect to see no differences in TFBS footprinting in Hira KO vs WT ESCs? Please clarify.
We actually do find that HIRA is involved in H3.3 deposition at regulatory elements, while ATRX and DAXX globally are not. We plotted H3.3 ChIP-seq data from WT and chaperone KO ESCs (HIRA, ATRX, or DAXX) and found that H3.3 deposition is reduced at enhancers and promoters in HIRA KO ESCs only. In agreement with potential competition between the chaperone systems, we see that H3.3 deposition at enhancers and promoters is modestly increased in ATRX and DAXX KO ESCs. These data are now included as Supplemental Figure 2.

9. Results from the TFBS footprinting analysis suggest reduced binding of nearly all 395 expressed TFs analyzed, which makes one question whether results from this analysis are reliable. To strengthen this analysis, the authors should consider ChIP-Seq or ChIP-qPCR analysis of a handful of TFBSs overlapping H3.3 peaks showing reduced binding in KO cells; as negative controls, include a set of TFBSs not overlapping H3.3 peaks (and/or not showing reduced binding).

We have now included TF CUT&Tag for KLF5 and SOX2, showing reduced binding in H3.3 KO ESCs compared to WT. These data are included as Figure 2E and Supplemental Figure 9E-F.

10. Base on Fig. 3 data, the authors argue that H3K4me3 at active promoters has "little reliance" on H3.3 whereas p300, H3K27ac, and BRD4 exhibit "reduced enrichment" although the p-value for the reduced signal in all four cases is <2.2x10E-6. Not sure if this conclusion is justifiable. Please clarify. Also include density profiles for H3K4me3, p300, H3K27ac, and BRD4 (as in Fig. 1C and 2B).
The reviewer is correct that H3K4me3 levels are statistically different between WT and H3.3 KO ESCs. What we meant to say is that H3K4me3 levels seem less affected compared to the acetyl machinery, as evidenced by a smaller fold-change between WT and H3.3 KO samples (see the box plot and the density plot showing direct region comparisons in Figure 3). We have now clarified our description of these data sets in the text.
We now include density profiles for H3K4me3, p300, H3K27ac, and BRD4 in Figure 3.

11. Fig. 1E: please also include box plots for all promoters (Fig. 1C).
Boxplots for all active promoters are now included in Fig. 1E for H3.3 KO, and Supplemental Figure S3 for chaperone KO ESCs.

12. Fig. S2B and related text: please consider adding a Venn diagram showing the overlap between H3.3 dependent promoters, HIRA-dependent promoters, and ATRX/DAXX-dependent promoters.
This is an important suggestion, to ask whether changes in chromatin accessibility are correlated between H3.3 loss and HIRA loss, but not between H3.3 loss and ATRX or DAXX loss. We now provide these analyses at all active promoters identified in our study. Overall, we find that changes in chromatin accessibility at promoters observed after H3.3 KO are positively correlated (rho = 0.316) with those observed after HIRA KO. We find no correlation between H3.3 loss and ATRX or DAXX loss (rho = 0.07, -0.04 respectively). These analyses are included as a new Supplemental Figure S3H-J.

Minor points:
13. Fig. S3: panels B, D, F, H missing p-values.
These have been added.

14. Line 170-172, "Loss of TF binding ..." should be "Reduced/decreased TF binding". Similarly, "global loss of TF expression" should be "decrease in TF expression"

These changes have been made.

Reviewer #2: The functional role of histone H3.3 is not fully understood, and its pleotropic effects have been revealed in various types of genomic regions, possibly due to its role as a fundamental structural element of chromatin templates. In this manuscript, Tafessu and colleagues describe a series of phenotypes associated with TSS regions in H3.3 KO mESCs. Using epigenome profiling techniques including ATAC-seq and ChIP-seq, the authors show that in H3.3 KO mESCs, the average of chromatin accessibility in TSS regions is mildly reduced, along with a concomitant decrease of histone modifications and transcriptional co-activators all of which are associated with active transcription. By performing GRO-seq, the authors revealed that nascent RNA levels around TSS region were also reduced in H3.3 KO mESCs, further suggesting a role for H3.3 in active gene transcription. Since mESCs lacking H3.3 showed minor defects in gene expression as measured by RNA-seq, the authors then used ATAC-seq to show that TF bindings is impaired during mESC differentiation in H3.3 KO EBs. Finally, they propose a function of H3.3 in facilitating TF binding at newly active promoters.
Overall, the conclusions are not convincing enough, especially the TF binding part. The differences described for the promoter regions between WT and H3.3 KO are relatively mild and most analyses are quite crude. Moreover, I am concerned about the observation of ATAC-seq signal loss at the TSSs after losing H3.3 and its interpretation. I suspect the minor alterations observed in the study is caused by normalization.
Major Point:
1. The authors revealed a trend of decrease in ATAC-seq signal at the TSS regions, yet no such trend at enhancer regions. Enhancers are also enriched for TFs and H3.3, it is hard to reconcile their results with their model.
We have clarified this point. We do see a small decrease in ATAC-seq signal at enhancers that is not as obvious as the decrease that we see at promoters, see new data added in Supplemental Figure 1. We’re not sure why the effect is more pronounced at promoters. We do see that more enhancers are detected as differentially accessible by DESeq, but there is no directionality to the response (see Supplemental Figure 5). Maybe this masks the effect to some extent?
We feel confident in our results, based on the comprehensive analyses we provide at both genes and repeat regions (see below) in H3.3 KO ESCs and all chaperone KO ESCs. Further, the TF CUT&Tag (new Figure 2E), ChIP-seq, and GRO-seq results follow as expected from the ATAC- seq, giving additional credence to our interpretations.

2. The authors suggest that TF binding at the TSS is also compromised due to loss of H3.3. It is quite counterintuitive that chromatin becomes less accessible, when both TFs and histones are locally reduced.

We agree that this result is somewhat counterintuitive, if the role of H3.3 in ESCs is to provide a
nucleosome at these regions. We think that the result in this cell type may have something to do with cell cycle. ESCs are dividing every 12-18 hrs and spend about 70% of this time in S phase. As such, they have ample access to canonical histone H3.1/2, which would be deposited during replication and may lead to increased nucleosome occupancy at the regions in question. Our ATAC-seq data support this interpretation. To further address this question, we now provide analysis of general H3 ChIP from WT and H3.3 KO ESCs. In support, we find that overall levels of H3 enrichment are slightly higher at promoters in H3.3 KO ESCs compared with WT ESCs. These data are now included as Supplemental Figure S3K.

3. The authors should analyze the ATAC-seq signal across the genome, to address whether their observed accessibility loss is an indirect consequence of normalization. This is particularly important because an increase of ATAC-seq signal at repetitive elements has been reported before (e.g., Carmen Navarro et al., 2020). During data processing, sequencing reads were normalized to the same depth for comparison. Is the small differences at the TSS regions a secondary effect of data normalization?
We don’t think that the effects we observe are a secondary effect of data normalization. Perhaps the best way to demonstrate this data is to analyze the repeats, as the reviewer suggested. Since much of this data has been published, we focused on IAPEz only. It was previously reported that accessibility at this element increases in H3.3 KO ESCs (actually using our published ATAC-seq data). We are able to reproduce this analysis. So, we find that loss of H3.3 decreases accessibility at active promoters and increases accessibility at IAPEz. Next, we checked IAPEZ accessibility in each of the chaperone KO ESCs. As predicted, IAPEz are more accessible in both the DAXX KO and ATRX KO ESCs. Importantly, they remain closed in the HIRA KO ESCs, where H3.3 deposition at ERVs remains intact. To summarize:

H3.3 KO promoters are less accessible	| ERVs are more accessible
HIRA KO promoters are less accessible	| no change at ERVs
ATRX KO no change at promoters	| ERVs are more accessible
DAXX KO no change at promoters	| ERVs are more accessible
These data are included as a new Supplemental Figure 6.

4. The authors claim that the ATAC-seq peaks decrease at TSS regions. However, as shown in Fig. S2B, both increase and decrease of ATAC-seq signal can be detected at different TSSs. What is the difference between the two groups with different response?
We have now profiled these promoters for ChIP-seq enrichment of H3K4me3, H3K27ac, H3K27me3, and H3.3. Generally, we find that the promoters that rely on H3.3 for accessibility carry a signature of activity (i.e., show higher enrichment of H3K4me3 and H3K27ac) while the promoters that become more open with H3.3 loss are more enriched with H3K27me3. One

interpretation of this result is that highly active genes rely on H3.3 to promote open chromatin, while repressed genes rely on H3.3 for PRC2 recruitment, as previously published (PMID 24074864 Banaszynski et al Cell 2013). These data are included as a new Supplemental Figure 5 and we have modified the text to present this more balanced view. To try to understand why these promoters show differential response, we performed motif search from each group. However, we found the most enriched motifs for each group were the KLF and SP family TFs (likely due to high GC content of promoters), and there were no motifs identified that were unique to either group.

5. By analyzing ATAC-seq data at the EB stages, the authors suggest that H3.3 KO may affect TF binding at novel binding sites during differentiation. What are the gene expression levels of differentiation-specific TFs in H3.3 KO EBs? If these TFs also show differential expression level, I cannot agree with the authors, instead it can simply reflect a global defect in differentiation. For example, the authors attempted to claim that H3.3 deposition is essential for establishing new TF binding patterns during differentiation, as lineage-specific TFs showed greater binding in WT EBs (Fig 5B). However, HAND2, GATA3, GATA2, TWIST2 was not even activated in H3.3- loss EBs, evidenced by RNA-seq data they used. Similarly, pluripotency-maintaining TFs (KLF5, KLF4, NANOG, POU5F1, ZFP43 et. al) were not silenced in the absence of H3.3 during EB differentiation. Therefore, the expression of corresponding TFs, but their chromatin binding, is critical for differentiation.
This is a really good point. First, we have identified TFs that show differential expression between WT ESCs and EBs (log2 FC > 0.5, p-value < 0.05). We next generated a heatmap showing expression of these genes in WT ESCs and EBs and H3.3 KO ESCs and EBs (included as a new Supplemental Figure 13F). These data show that while some EB-specific TFs are upregulated in H3.3 KO EBs (labeled on the heatmap), many are not. This confounds the analysis, as pointed out by our reviewer.
To more directly address the question of a role for H3.3 in TF binding, we have added a new experiment. Here, we inducibly express (using a Tet-on system integrated using a Piggybac plasmid) differentiation-specific TFs in both WT and H3.3 KO ESCs. Even though the TFs are expressed to similar levels in both WT and H3.3 KO ESCs, we observe reduced enrichment on chromatin in the absence of H3.3. These data suggest that H3.3 promotes de novo TF binding and are included as a new Figure 6.

6. The authors identified only 265 regions with lower accessibility in H3.3-depleted mESCs (Fig S2A). However, it seems that p300 and BRD4 recruitment at all promoters was disturbed (Fig 3, n=12,903), and the same is true for GRO-seq signal at promoters of expressed genes (Fig 4A). Any reasonable explanation?
Our analysis of all active promoters in ESCs shows that loss of H3.3 is correlated with reduced chromatin accessibility at promoters, as shown in Figure 1. This observation is phenocopied in HIRA KO ESCs but not in ATRX or DAXX KO ESCs, as shown in Supplemental Figure 3. These

data are very much in agreement with the global reductions in H3K4me3, p300, H3K27ac, and BRD4 that we report in Figure 3. It is true that only 265 regions are identified from DE analysis. This is likely due to the relatively modest effect of H3.3 loss on chromatin accessibility and the statistical cut-offs in place for our DE analysis.

7. It is surprising to see that only 1636 promoters were enriched with p300 (Fig S6B).
We agree, as we expect that all expressed promoters will recruit p300 to some degree. As the reviewer can see, the p300 data set used in these analyses is not as high quality as other data sets. We have had trouble with this experiment since loss of the Santa Cruz polyclonal rabbit antibody and would be very open to hearing whether there is a replacement antibody for these experiments.

Minor Point
1. line 120-122. As shown in Fig S2B, the number of differential ATAC-seq peaks at enhancer regions are in fact more than those at TSS regions. It is not appropriate to claim that there is NO change at the enhancer regions.
We now present a more balanced view of the changes we observe and enhancers and promoters, with improved analyses in Supplemental Figures 1 and 5 and modified text throughout, including a change in title.

2. CTCFL is not expressed in mouse ES cells. It does not make sense to include its binding motif in Fig 2C.
Our threshold for expression was basemean of >20 across WT and H3.3 KO RNA-seq data. The value for CTCFL expression from our data was 34. Given that its motif was identified computationally, we do not think that it is fair to exclude it.

3. The total number of promoters and enhancers should be indicated in the figures.
We have updated the text, figures, and figure legends to be sure this information is included for all analyses.

Reviewer #3: The histone H3 variant, H3.3, is essential for development, as knockout of H3.3 in mice is lethal due to severe developmental defects. Hence, understanding the detailed mechanism of how H3.3 regulates transcriptional programs in development is of great interest and significance.
The manuscript by Tafessu et al., specifically focused on the chromatin accessibility of promoter-proximal regulatory regions upon loss of H3.3, then characterized the related chromatin status in terms of H3K27ac, its writer (p300) and reader (BRD4) as well as RNA ploymerase, finally defined footprintings of ESC- and lineage-specific TFs accounting for the diminished capacity of H3.3 KO mESC to differentiate. This is an extended study from their previous work (Martire et al., Nat Genet, 2019) where they reported that the H3.3-regulated enhancers, together with H3K27ac and p300 are involved during mESC differentiation.
Compared to their previous study, they provided new proofs from the perspective of H3.3-bound promoters to explain the compromised ability of H3.3 KO mESC to differentiate. The new findings in the present study include: i) the H3.3-bound promoter regions are characterised; ii) they integrated RBD4 and RNA polymerase into the picture; iii) the footprintings of ESC- and lineage-specific TFs are defined. However, this study, in its current form, did not provide very convincing data, due to the lack of the validation experiments and the weak/very mild correlation between the high-throughput sequencing data.
The following are my concerns.
Major concerns:
1. In Line 131, the authors claimed that "In agreement with previous studies, we find that H3.3 enrichment at active regulatory elements is correlated with accessibility", as shown in Fig. 1A and Fig. S1A. However, the correlation coefficient (ρ) showed only 0.463 and 0.2 for promoters and enhancers, respectively, indicating mild or no correlations. Also, in Figure D, the correlation (ρ=-0.194) seems too weak to support the statement "Globally, we find that reduced ATAC-seq signal at promoters in H3.3 KO ESCs is correlated with H3.3 enrichment in WT ESCs (Line 125)". The same for Figure 4B (ρ=0.38) as well as Fig. S6 F (ρ=-0.248), G (ρ=-0.373) and H (ρ=-0.358). The authors should avoid being over-confident in their conclusions.
As noted, the Pearson correlation coefficients we calculate are modest. We could not find agreed upon terms to describe data sets within a range of correlation coefficients, which is why we simply used the term “correlated”. Overall, given the large amount of orthogonal genomics data sets arriving at the same conclusion of H3.3-depenence, we are confident in our interpretation that H3.3 helps shape the chromatin landscape at promoters. Also, while the correlation coefficients are modest, the p-values are not, suggesting that our observations are statistically significant.

2. Concerning the H3.3 ChIP-seq data (Figure 1A and 1D), could the authors show the global heatmap and some representative examples? Also, did the authors perform ChIP-seq under H3.3 KO condition, together using IgG antibody as negative controls in order to obtain H3.3 peaks of high confidence?
The data sets used in this paper were generated over the course of previously published studies. In PMID 31152160 (Martire et al, Nature Genetics 2019) and PMID 25938714 (Elsasser et al, Nature 2015) , we present global heatmaps of H3.3 enrichment and representative browser tracks demonstrating specific enrichment. In PMID 25938714, we performed ChIP using the H3.3 antibody from both WT and H3.3 KO ESCs, demonstrating the specificity of the antibody. For ease of reviewer assessment, we include these published data here. Panels A-D are from Elsasser et al, and Panels E-G are from Martire et al

3. In Fig. 1B, C and Fig. S1B, the authors observe a slight but significant decrease in chromatin accessibility at promoters but not distal regulatory elements (enhancers) in the absence of H3.3. However, in Supplementary Figure 2A, it shows that the number of differentially accessible enhancers is more than that of promoters in H3.3 KO compared to WT ESCs, indicating that the accessibility in both enhancers and promoters is changed. Could the authors explain this?
We have gone back to these analyses to consider them more carefully. It is true that the average profiles of ATAC-seq data at enhancers show little global change while reduced ATAC signal in H3.3 KO ESCs is visibly appreciable at promoters. This is inconsistent with the DiffBind analysis that identified several hundred up and downregulated enhancers each. To directly compare ATAC-seq signal at individual loci, we generated density plots, plotting the ratio (log2) of signal from H3.3 KO / WT ESCs. This representation shows a modest reduction in accessibility at enhancers in the absence of H3.3 that is statistically significant. These data are included in a new Supplemental Figure 1. We still do not know why the effect is more pronounced at promoters. We have updated the text to be more reflective of these results throughout, including a change in title.

4. As shown in Figure 4 A and B, the binding of key TFs e.g. POU5F1, NANOG, SOX2 are differentially changed, together with the pol II engagement at promoters upon depletion of H3.3, although the global transcription remains undisturbed in ESC stage. Also in Figure 2B, the reduction in promoter binding by e.g. OCT4 and NANOG seems very minor. Therefore, some top candidates genes/well-known OCT4/NANOG target even their own promoters should be assessed by e.g ChIP-qPCR, given that POU5F1, NANOG, SOX2 forms an interconnected autoregulatory circuit, by targeting both their own promoters and those of each other. Similar experiments should be applied to the EBs in terms of lineage-specific TFs.
We agree with the reviewer that it is important to directly assess TF binding. We have performed CUT&Tag genome-wide profiling of KLF5 and SOX2 occupancy in WT and H3.3 KO ESCs. These data demonstrate that these TFs show reduced binding in the absence of H3.3. These data are included as a new Figure 2E and Supplemental Figure 9E-F.
We agree that EB TF ChIP-seq is also an important experiment. We had difficulties isolating nuclei / chromatin for this experiment due to the small numbers of cells that we typically take into an EB assay. To more directly address the question of the importance of H3.3 for new TF binding, we now present an alternative experiment. Here, we express differentiation-specific TFs in WT and H3.3 KO ESCs using a Tet-on system. If H3.3 is required for new TF binding, we should see lower levels of chromatin occupancy in H3.3 KO ESCs, even when the new TF is expressed at the same level in both WT and H3.3 KO ESCs. This is exactly what we observe. We include this new experiment for three differentiation-specific TFs as a new FIgure 6.

5. In Supplementary Figure 7, the authors showed the transcriptional changes with loss of H3.3 in ESCs and during differentiation. However, the comparison between H3.3KO EBs and WT EBs is missing.
We have now added a comparison between H3.3 KO EB and WT EB RNA-seq as Supplemental Figure 13E-F. We find that many pluripotency factors (e.g., Oct4, Nanog, Dppa2, Zfp42, etc.) show greater expression in H3.3 KO EB compared to WT EB, suggestive of a differentiation delay or defect in H3.3 KO cells. Further characterization of WT and H3.3 KO EBs can be found in the Supplemental Material of PMID 31152160 (Martire et al, Nature Genetics 2019) and included in a response to the previous reviewer.

6. Concerning the differentiation strategy, the authors employed the undirected differentiation (Line 35 and 36) to evaluate the effect of loss of H3.3. In this way, the heterogeneity in the cell population might make the data interpretation difficult and inaccurate. Perhaps directed differentiation would be better. The authors are encourage to discuss this in the discussion section.
We agree that directed differentiation would be an effective way to enforce broader specificity of lineage TF expression in the differentiating cells, and that the undirected nature of EB differentiation may mute the observed differences between lineage TF binding in WT and H3.3 KO cells. We now include this as a point of consideration in the discussion.

Minor concerns:
1. In Figure 3A, for the first panel, the Heatmaps of ATAC-seq in both WT and H3.3KO should be shown for a better comparison.
These heatmaps have been added.

2. In Methods part, the mES cell line (Line 366) should be specified.
This information has been added.

3. Regarding the differentiation protocol, did the authors adopt it from a literature or not? How efficient is the protocol? Did they analyze any marker genes?
This protocol was carried out as described and reported in PMID 31152160 (Martire et al, Nature Genetics 2019). In this publication, we analyzed marker genes by qPCR and western blot. These data show that pluripotency markers such as Oct4, Sox2, and Nanog were downregulated over the course of EB differentiation of WT cells, but not in H3.3 KO cells. We also showed upregulation of lineage markers from endoderm (e.g., Gata2, Gata4), ectoderm (Cdx2, Otx6), and mesoderm (e.g., T, Fgf5) in WT cells that was attenuated in H3.3 KO cells. We also performed RNA-seq analysis that was included in Supplemental Figure 7 of this paper. This figure is included below for reviewer ease.

4. In Supplementary Figure 3, the exact P values should be shown either in figure or in figure legend.
P values have been added to what is now Supplementary Figure 4.

Reviewer #4:
Title: H3.3 Contributes to chromatin accessibility and transcription factor binding at promoter proximal regulatory elements.
Summary of Results: Histone variant H3.3 has been established to be present at dynamically regulated portions of chromatin such as promoters, gene bodies, and enhancers. However, the exact function of H3.3 in maintaining the proper accessibility and function of these regions is not fully understood. In this study, the authors identify promotor dysregulation in the absence of H3.3 in mouse embryonic stem cells. This decreased accessibility corresponded to decreased TF foot printing (reduced accessibility to chromatin binding by TFs?) & dysregulation of the chromatin landscape in these cells (secondary to reduced TF binding?), without dramatically changing transcriptional output (heterogeneity and selection of ESC with reduced transcriptional defects?). However, the authors show decreased RNAPII engagement in the TSS of expressed genes, which implies that proper TF binding and RNAPII engagement requires promotor proximal H3.3. What the mechanism is that links H3.3 deficiency to reduced TF binding and RNAPII activity is unclear and yet may be readily illuminated with minimal additional effort, strengthening the paper and its utility to the field.
The manuscript is scholarly and well written and in general the conclusions derived from the data are sound. It is an important contribution to the H3.3 field and an understanding of this important histone variant in transcription. However, it is critical for interpretation and mechanistic understanding of the phenotypes described for the authors to perform experiments, analysis, and provided discussion of how nucleosome occupancy is changing following H3.3 KO (see point 1, below).
Major Comments:
1) Is H3.1/2 is localized to regions normally occupied by H3.3 in the H3.3 KO cells? ES cells are rapidly cycling and S-phase expressed H3.1/2 could be occupying the nucleosomes at these regions. Thus, since H3.1/2 is generally considered to me more stable in a nucleosome with H2A.Z than H3.3, could this explain the decreased accessibility, reduced TF binding, with transcriptional changes. To address this H3.3 and a H3.1/2 ChIPs (and/or pan H3) should be performed in WT/KO cells. Additionally, H2A.Z ChIPs would be interesting but are not necessary for interpretation of the main point of nucleosome occupancy, but rather go beyond to address composition and classes of promoters that may be regulated by H2A.Z/H3.3 nucleosomes.
This is an excellent suggestion from the reviewer, that rapidly dividing ESCs have ample access to replication-coupled H3.1/2 that may be occupying the regions in question in the absence of H3.3. To test this, we now provide analysis of pan-H3 ChIP-seq (using Abcam antibody Ab1791) in WT ESCs compared to two H3.3 KO ESC clones. These data suggest that promoters are enriched with higher levels of H3.1/2 in the absence of H3.3 and are included as a new Supplemental Figure S3K. We also make note of this point in the discussion.
We also agree that the point of distinct classes of promoters that may be regulated by H2A.Z/H3.3 nucleosomes is an important consideration, given past literature on nucleosomes of this composition, and is deserving of additional study in the future.

2) The data that reveal regulation of H3.3 deposition by HIRA complex vs ATRX/DAXX are interesting. However, the conclusions would be strengthened by ChIP for H3.3 in chaperone knock out experiments. Does H3.3 deposition increase in DAXX KO at some loci? To what extent does H3.3 deposition decrease in HIRA KO? HIRA complex could also have function in local H3.3 turnover and activities on H3.3 bound chromatin independent and beyond function in H3.3 deposition.
We now include analyses of H3.3 ChIP-seq data from chaperone KO ESCs. Generally, we find that H3.3 deposition decreases at both enhancers and promoters in HIRA KO ESCs. In agreement with a model in which chaperone complexes compete for H3.3 substrate, we find a

subtle increase in H3.3 deposition at regulatory elements in both ATRX and DAXX KO ESCs.
These data are included as a new Supplemental Figure 2.
The reviewer is absolutely correct that the HIRA complex could also function in local H3.3 recycling in addition to H3.3 deposition, as recently demonstrated by the Almouzni lab. Our analysis are not in conflict with the idea of recycling, and this is an important consideration for future study.

3) The GRO-seq experiment in Figure 4 provides an interesting insights but the authors don't follow up (experimentally or with sufficient discussion of possible interpretations) on any potential mechanism that is causing the reduced engagements of RNAPII with promoters in the absence of H3.3. While "TF footprint" analysis suggests reduced TF binding, the computational approaches employed are not strictly footprinting analysis, rather motif-proximal/associated accessibility. To infer chromatin binding by TF more directly the authors should employ other tools dedicated for this kind of analysis, for example HINT: https://doi.org/10.1186/s13059- 019-1642-2. Such analysis could improve these interpretations. However, moving beyond TF binding and links to RNAPII recruitment and activity as interpreted by GRO-seq, the authors only provide superficial GRO-seq data visualization and analysis. Can they show some example tracks of these data with characteristic reductions in 5' GRO-seq signal in H3.3 KO cells? Can they interpret ratio of 5' to 3' GROseq to make inferences such as that H3.3 KO is reducing RNAPII initiation but not reducing elongation rates, etc?
We now include the suggested analysis looking at specific TF cutsite data in a new Supplementary Figure 7.
We have now included GRO-seq average profile of active promoters in ESCs, as well as pause index assessment. These data are included as a new Supplementary Figure 12. They suggest a reduction both in RNAPII initiation as well as elongation rates. We have not performed appropriate experiments to draw any conclusions about how H3.3 may be affecting each process, which we leave for future studies.

Minor Comments:
1) What about ERVs in ESC? The last author previously published on H3.3 enrichment at ERVs and recent studies have further highlighted that H3.3 may regulate ERV expression, perhaps linked to bivalent chromatin (https://www.nature.com/articles/s41556-021-00795-7). Do the authors relate these results to the ERV de-repression and nucleosome depletion/accessibility at these regions.
We now include analysis of the IAPEz ERV in all cell lines used in this study, please see response to reviewer above and Supplemental Figure 6. In short, ERVs become more open in H3.3 KO, DAXX KO, and ATRX KO ESCs, but are unaffected in HIRA KO ESCs. We certainly agree that H3.3 through the ATRX/DAXX pathway plays an important role in regulating ERVs under a number of physiological settings.

2) The authors interpret their results to say that H3.3 is necessary for nucleosome
displacement to occur. But the paradox of deleting H3.3 potentially leading to nucleosome depletion is not addressed. These ideas—nucleosome depletion vs replacement with H3.1/2 nucleosomes should be discussed and presented clearly for readers to accurately interpret the results presented.
This is an important point that we now address with the addition of pan-H3 ChIP in H3.3 KO (see response to reviewer above) included as a new Supplemental Figure S3K, and also in the discussion. We think that this may be a feature of rapidly cycling cells such as the ESCs used in this study.

3) The authors don't mention all the antibodies they used for ChIP-seq (i.e. anti-H3K27ac) or show any antibody validation data (for example equivalent binding of K27ac on H3.3 vs H3.1/2 is critical—if data is available, great, if not, it is critical for the specific reagent to be specified and any available specificity data presented).
The data sets analyzed in our current study were previously published and deposited to GEO (see PMID 31152160 - Martire et al, Nature Genetics 2019 - GSE114551). All data sets used for each figure in the current submission can be found in Supplemental Table 4.
We had the same concerns as this reviewer regarding the specificity of the H3K27ac antibody, in that perhaps what looks like a reduction of H3K27ac in H3.3 KO ESCs is simply due to an antibody that binds to H3.3K27ac better than H3.1/2K27ac. To assuage these concerns, we first used H3.1K27ac and H3.3K27ac peptides in dot blot assays and found that both peptides were equally recognized by the H3K27ac antibodies used in our studies (AM39133 polyclonal rabbit and AM39865 monoclonal mouse). Further, we performed mass spectrometry, where we found that H3K27ac was reduced on H3.1/2 in H3.3 KO ESCs. This data can be found in Figure 1 and Supplementary Figure 1 of PMID 31152160. For ease of reviewer analysis, we also include this published data here

4) The subfigures in Figure 3 seem somewhat redundant as the heatmap, boxplot, and density plot are all conveying same message of decreases these active histone marks & their cofactors at promoters in H3.3 KO ES cells. What about the promoters (and associated genes) where this effect is the most drastic? Is this affect the same genome wide or a similar trend to the change in accessibility where the promoters with the most H3.3 in WT cells have the greatest decrease in H3K27ac, p300, etc.?
We agree that these representations are somewhat redundant, but they do serve various purposes, with (1) average profiles providing a “quick glance” interpretation of the data, (2) heatmaps demonstrating whether the effects are driven by outliers, (3) boxplots allowing statistical comparisons, and (4) density plots allowing direct comparisons between enrichment loci that are lost in the box plot.
We do see that the level of H3.3 deposition at a promoter is generally correlated with decreased enrichment of H3K27ac, p300, etc., in H3.3 KO. These data are included as Supplemental Figure 11E-H. Additionally, we find that the regions we computationally identified as “H3.3-dependent” from our ATAC-seq DE analysis display a more substantial reduction of chromatin proteins / marks profiled. These data are included as a new Supplemental Figure 11I-L.

5) Why aren't HIRA, ATRX, & DAXX KO ES cells used in the differentiation experiment in Figure 5 as a comparison to H3.3 KO (as they are for much of the rest of the study)?
This is an excellent point. We have tried differentiating all chaperone KO ESCs into EBs, and all show some failure or defect in differentiation. We have not studied the precise mechanisms, but we presume that the ATRX and DAXX KO EBs fail due to repeat element dysregulation. A more careful assessment is certainly important for future studies.
To more directly address the question of a role for H3.3 in new TF binding, we have provided an alternative experiment. We inducibly express differentiation-specific TFs in WT and H3.3 KO ESCs. These TFs, that the cells have not seen before, are expressed at similar levels in both WT and H3.3 KO ESCs. Despite that, we find that these TFs show reduced enrichment on chromatin in the absence of H3.3, suggesting that the histone variant promotes TF binding. We show this for three different TFs. These data are included as a new Figure 6.

**2^nd^ round**

**Reviewer 1**

The authors have satisfactorily addressed all of my comments.

**Reviewer 2**

The revised manuscript is significantly improved. Nevertheless, I have a few questions and comments to be addressed before publication

1. As a summary of the main message of this study, the old title is more balanced than the new one.

2. Fig 6, the authors include a new experiment to suggest that H3.3 is important for initiation of new transcriptional programs. How do these TF peaks associate with the ATAC-seq peaks in ESCs? I think the interpretation of the result would become confounded if these TFs turn out to prefer active promoters that are affected by H3.3, as in this situation, the behaviors of the ectopic TFs show little difference to the pluripotent-associated TFs. Furthermore, to strengthen their claims, the authors are encouraged to perform gene expression analyses to confirmed that H3.3 can influence gene expression mediated by lineage-specific TFs.

3. A recent report has raised concerns on the pA-Tn5-based epigenome profiling methods. It is suggested that high levels of false positive signals overlapping with ATAC-seq may be recovered if assay conditions are not optimized (https://doi.org/10.1101/2021.07.09.451758). To avoid systematic biases of their CUT&Tag data towards active promoters, can the authors verify that their CUT&Tag profiles are TF-specific such as that the peaks can indeed enrich the motifs of the corresponding TFs.

**Reviewer 3**

The authors have addressed most of my concerns and the manuscript is much improved. Therefore, I have no additional questions.

**Authors’ response**

Reviewer #2: The revised manuscript is significantly improved. Nevertheless, I have a few questions and comments to be addressed before publication

1. As a summary of the main message of this study, the old title is more balanced than the new one.

We have reverted to the old title, “H3.3 contributes to chromatin accessibility and transcription factor binding at promoter-proximal regulatory elements”.

2. Fig 6, the authors include a new experiment to suggest that H3.3 is important for initiation of new transcriptional programs. How do these TF peaks associate with the ATAC-seq peaks in ESCs? I think the interpretation of the result would become confounded if these TFs turn out to prefer active promoters that are affected by H3.3, as in this situation, the behaviors of the ectopic TFs show little difference to the pluripotent-associated TFs. Furthermore, to strengthen their claims, the authors are encouraged to perform gene expression analyses to confirmed that H3.3 can influence gene expression mediated by lineage-specific TFs.

This is an interesting point. We have now compared peak calls at promoters from our ATAC-seq and de novo expressed TF CUT&Tag data. As suggested by the reviewer, we find that most of the TF signal is found within a subset of the ATAC-seq peaks found in WT ESCs. We do think this is specific, because the TF data sets are enriched with their expected motif. In addition, for each TF, we find several hundred TF peaks outside of pre-existing open chromatin. These regions are bound with similar intensity as those within already accessible regions in WT ESCs and are also sensitive to H3.3 loss. We now include these data as a new **Supplementary Figure 14**. We propose that this likely has biological relevance, in that newly expressed TFs prefer to bind previously accessible chromatin, meaning that H3.3 KO-induced accessibility loss in ESCs will almost certainly affect how well new TFs expressed during differentiation are able to bind. In other words, not only would differentiation TFs be less able to bind because of direct effects of H3.3 loss, but they also would have less accessible chromatin to bind to because of accessibility that was lost before differentiation.

As the reviewer states, if H3.3 is playing a role in gene expression mediated by lineage-specific TFs, we should see evidence of this in transcriptomics data from this experiment. While this experiment would improve our study, it would not significantly alter our conclusions and we ask to resubmit without its inclusion.

3. A recent report has raised concerns on the pA-Tn5-based epigenome profiling methods. It is suggested that high levels of false positive signals overlapping with ATAC-seq may be recovered if assay conditions are not optimized (<https://doi.org/10.1101/2021.07.09.451758>). To avoid systematic biases of their CUT&Tag data towards active promoters, can the authors verify that their CUT&Tag profiles are TF-specific such as that the peaks can indeed enrich the motifs of the corresponding TFs.

This is a valid concern. As the reviewer suggests, we have verified that CUT&Tag profiles are TF-specific. Using HOMER, we find that the KLF5 data set is highly enriched with KLF motifs while the SOX2 data set is highly enriched with SOX motifs. We also include motif enrichment from each data set at promoters and IAPEz elements as a new **Supplementary Figure 9G and 9H**.

Finally, a big thank you to our editor and reviewers for improving the quality of this work!

**3^rd^ round**

**Reviewer 2**

No further comments, congrats!
